# Mechanisms of Metabolic Reprogramming in Cancer Cells Supporting Enhanced Growth and Proliferation

**DOI:** 10.3390/cells10051056

**Published:** 2021-04-29

**Authors:** Chelsea Schiliro, Bonnie L. Firestein

**Affiliations:** 1 Cell and Developmental Biology Graduate Program and Department of Cell Biology and Neuroscience, Rutgers, The State University of New Jersey, 604 Allison Road, Piscataway, NJ 08854, USA; css161@scarletmail.rutgers.edu; 2 Department of Cell Biology and Neuroscience, Rutgers, The State University of New Jersey, 604 Allison Road, Piscataway, NJ 08854, USA

**Keywords:** Warburg Effect, cancer, oxidative phosphorylation, aerobic glycolysis, pentose phosphate pathway, one-carbon metabolism

## Abstract

Cancer cells alter metabolic processes to sustain their characteristic uncontrolled growth and proliferation. These metabolic alterations include (1) a shift from oxidative phosphorylation to aerobic glycolysis to support the increased need for ATP, (2) increased glutaminolysis for NADPH regeneration, (3) altered flux through the pentose phosphate pathway and the tricarboxylic acid cycle for macromolecule generation, (4) increased lipid uptake, lipogenesis, and cholesterol synthesis, (5) upregulation of one-carbon metabolism for the production of ATP, NADH/NADPH, nucleotides, and glutathione, (6) altered amino acid metabolism, (7) metabolism-based regulation of apoptosis, and (8) the utilization of alternative substrates, such as lactate and acetate. Altered metabolic flux in cancer is controlled by tumor-host cell interactions, key oncogenes, tumor suppressors, and other regulatory molecules, including non-coding RNAs. Changes to metabolic pathways in cancer are dynamic, exhibit plasticity, and are often dependent on the type of tumor and the tumor microenvironment, leading in a shift of thought from the Warburg Effect and the “reverse Warburg Effect” to metabolic plasticity. Understanding the complex nature of altered flux through these multiple pathways in cancer cells can support the development of new therapies.

## 1. Introduction

Cancer is a complex genetic disease that arises from elaborate changes to the genome. This includes a cumulative collection of gain-of-function mutations that stimulate oncogenes, loss-of-function mutations that inactivate tumor suppressor genes, and mutations that inactivate stability genes involved in proliferative cell division, all of which help facilitate the transformation of a cell to a malignant phenotype. Characteristics typical of cell with a malignant phenotype include an unlimited ability to replicate, avoidance of apoptosis, insensitivity to anti-growth signals, continuous angiogenesis, self-sustained growth signals, tissue invasion, and metastasis [1]. In order for malignant cells to obtain the energy and materials required for acquisition and maintenance of these characteristics, they must undergo reprogramming of their metabolic pathways. Enhanced growth and proliferation via replicative division in cancer cells means they also require an increased amount of energy in the form of ATP, co-factors, such as NADPH and NADH, and building block molecules, such as carbon skeletons, to assemble new daughter cells. The increased demand for materials is satisfied through alteration of flux through key cellular metabolic pathways. Glycolysis and glucose metabolism are the most well-known altered metabolic pathways in cancer cells, with the first observations made about 100 years ago. Since then, there have been many other pathways found to be altered in cancer, such as glutamine metabolism, lipid acquisition, fatty acid oxidation, one-carbon metabolism, branched chain amino acid metabolism, and the citric acid cycle. The reprogramming of these pathways involves complex mechanisms and the coordination of a variety of signaling molecules, including molecules previously regarded as insignificant, non-coding RNAs. Furthermore, because of the complexity of these mechanisms, the reprogramming of metabolic pathways in cancer often occurs in various degrees and contexts in many types of cancer, affording cancer cells a plasticity that is not observed in normal cells. Much progress has been made in understanding the intricate nature of these pathways, providing a solid foundation for the development of new cancer therapies.

## 2. Glucose Metabolism and the Warburg Effect: A Century Later

Otto Warburg’s description of glucose metabolism in cancer cells was made almost a century ago, and it remains a key concept in the field of cancer metabolism. The Warburg Effect states that cancer cells rely on aerobic glycolysis (the conversion of glucose to lactate in the presence of oxygen) for ATP production, as compared to normal cells that rely on oxidative phosphorylation (OXPHOS) [2]. Since Warburg’s initial discovery, there has been a vast increase in knowledge of the role of aerobic glycolysis in cancer cells. More specifically, it has been found there is an increased ratio between glycolysis and oxygen consumption that is coordinated by changes to oxidative metabolism, the activation of oncogenes, and the loss of tumor suppressor genes [3]. The molecular mechanisms underlying the Warburg Effect are complex and involve many key molecular players.

**Figure 1:**
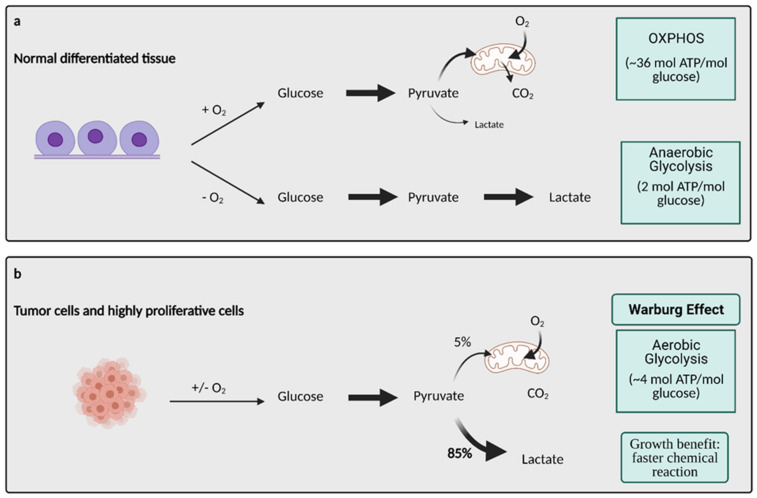
Glucose metabolism in normal differentiated tissue vs. tumor cells. (**a**) In normal differentiated tissues, one of two pathways is utilized. When oxygen is present, glucose is metabolized to pyruvate, which later enters OXPHOS to produce ~36 mol ATP/mol glucose. When no oxygen is present, glucose is metabolized to lactate, which yields 2 mol ATP/mol glucose. (**b**) Tumors and other highly proliferative cells prefer to convert the majority of their glucose to lactate to yield ~4 mol ATP/mol glucose, even in the presence of oxygen. This is called the Warburg Effect, and while it produces less ATP/mol glucose, it is a much faster chemical reaction than OXPHOS, conferring a major growth benefit to cancer cells. ATP; adenosine triphosphate, CO_2_; carbon dioxide, O_2_; oxygen, mol; mole. Figure created with BioRender.com (accessed on 26 March 2021).

Glucose metabolism is considered one of the most important aspects of cancer cell metabolism as it supplies intermediates and precursors for several other key metabolic pathways, including the generation of amino acids, nucleotides, and lipids [4]. Thus, one of the first questions posed about glucose metabolism in cancer is why cells would shift towards an energy generation mechanism that is less efficient. OXPHOS generates 36 ATPs per molecule of glucose while glycolysis only generates 2 ATPs for one glucose molecule [4]. Lactate generation, however, is an approximately two order of magnitude faster chemical reaction than OXPHOS, and therefore, offers a growth benefit (Figure 1) [5]. In order to obtain the uncontrolled growth that is characteristic of cancer, cells need to generate energy quickly, and this is accomplished effectively with aerobic glycolysis. The high output of lactate also generates an acidic microenvironment where only cells with phenotypes resistant to acidic environments can grow. This offers a huge growth advantage and intensifies the invasive and metastatic nature of cancer cells, as other cells around them deteriorate [6].

How exactly does a cell shift from OXPHOS to aerobic glycolysis? Over the years it has remained a highly researched area and has been found to involve elaborate mechanisms and molecules. Hypoxic conditions, resulting from inadequate vascularization, act as key initiator in the transition. As hypoxia increases in the tumor microenvironment, the cells employ certain stress responses as a means of survival [7]. These stress responses are mediated by oncogenes and tumor suppressors that activate specific molecules and signaling pathways that are key in regulating aerobic glycolysis. Some examples of these regulators are proto-oncogene Myc, transcription factor hypoxia inducible factor 1 (HIF-1), the PI3K/Akt/mTOR pathway, and tumor suppressor p53, all which are known to be expressed abnormally or altered in many different types of cancers [8]. Both the Myc and PI3K/Akt/mTOR signaling pathways are involved in cell growth and proliferation but have specific effects on glycolysis. Encoded by the Myc oncogene, transcription factor c-Myc upregulates multiple molecules involved in glycolysis, including glucose transporters (GLUTs), and glycolytic enzymes hexokinase 2 (HK2), phosphofructokinase (PFK), lactate dehydrogenase A (LDHA), and pyruvate dehydrogenase kinase 1 (PDK1). The PI3K/Akt/mTOR pathway increases the efficiency of glycolytic enzymes HK2 and PFK via Akt signaling [9]. Thus, both Myc signaling and the PI3K/Akt/mTOR pathway are hyperactivated in cancer cells. Tumor suppressor p53 naturally impairs glycolysis and favors OXPHOS by downregulating GLUT1, GLUT4, and HK2. The p53 gene is mutated in various types of cancers resulting in a loss of function, thus contributing to increased glycolysis [10]. 

**Figure 2:**
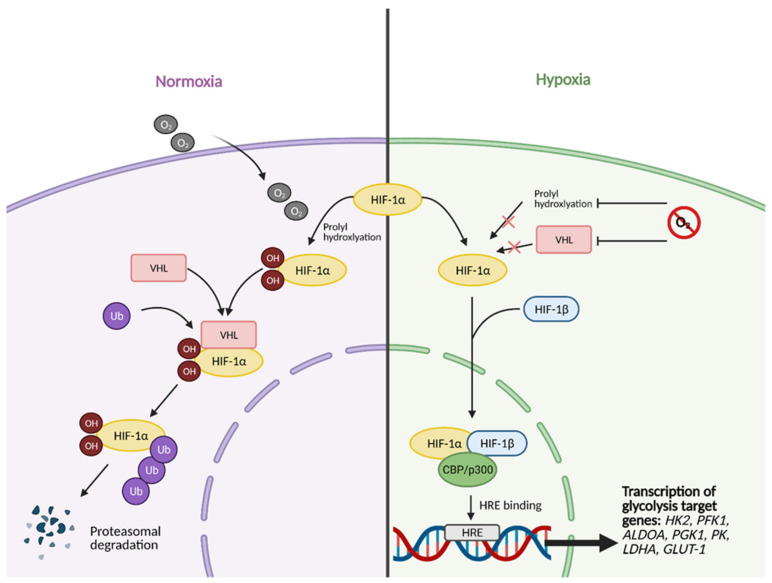
HIF-1α activation in normal vs. hypoxic conditions. Under normal conditions, in the presence of oxygen, the regulatory subunit of master transcription factor hypoxia-inducible factor (HIF-1), HIF-1α, undergoes prolyl hydroxylation, which induces HIF-1α binding to von Hippel-Lindau (VHL) tumor suppressor protein. This results in HIF-1α being tagged with Ubiquitin (Ub) to undergo proteasomal degradation. In hypoxic conditions, HIF-1α cannot undergo prolyl hydroxylation and subsequent binding to VHL. This allows it to bind to its partner, HIF-1β, and translocate to the nucleus. In the nucleus, fully functional HIF then forms a complex with transcriptional co-activators CBP/p300. The complex then binds to hypoxia response element (HRE) domains of the DNA, resulting in the transcription of several genes involved in glycolysis, such as HK2 (hexokinase 2), PFK1 (phosphofructokinase 1), ALDOA (aldolase A), PGK1 (phosphoglycerate kinase 1), PK (pyruvate kinase), LDHA (lactate dehydrogenase A), and GLUT-1 (glucose transporter 1). O2; oxygen, OH; hydroxyl group. Figure created with BioRender.com (accessed on 26 March 2021).

All of the aforementioned pathways and molecules have crosstalk with the master regulator and oxygen-sensing transcription factor HIF-1 [11]. Some examples include the activation of regulatory subunit HIF-1α by Akt and mTOR [12], the inhibition of Myc by HIF under hypoxic conditions and cooperation between the two molecules to promote cancer cell growth [13], and the inhibition of HIF-1α via high p53 expression [14]. This molecular crosstalk is especially important because HIF-1 exerts major effects on glucose metabolism when active. Unstable in highly oxygenic conditions, the regulatory subunit of HIF-1, HIF-1α, becomes stabilized under hypoxic conditions, allowing it to translocate to the nucleus and form a heterodimer with its binding partner, HIF-1β (Figure 2) [8]. This heterodimer binds to hypoxia-response enhancer sequence, hypoxia-response element (HRE) to induce the expression of multiple hypoxia-responsive genes [5]. HIF-1 in particular upregulates the expression of glycolysis enzymes hexokinase 2 (HK2), phosphofructokinase 1 (PFK1), aldolase A (ALDOA), phosphoglycerate kinase 1 (PGK1), pyruvate kinase (PK), and lactate dehydrogenase A (LDH-A) [5] and downregulates pyruvate dehydrogenase (PDH) activity via upregulation of PDH kinases (PDKs) to prevent transition into the citric acid cycle (TCA cycle) [15]. HIF-1 also upregulates the expression of other key molecules involved in aerobic glycolysis, including glucose transporters (GLUTs), such as GLUT-1 to increase glucose uptake and monocarboxylate transporters (MCTs), such as MCT4 for lactate transport out of cells [16]. Additionally, HIF-1 mediates the downregulation of OXPHOS through transcriptional activation of NADH dehydrogenase (ubiquinone) 1α subcomplex subunit 4-like 2 (NDUFA4L2), which inhibits Complex I of the electron transport chain (ETC) [17]. 

Recently, a “metabolic plasticity” theory of cancer cells has been described, where cells still have fully functional OXPHOS machinery and can switch between OXPHOS and aerobic glycolysis, or even perform them simultaneously [11]. This affords them the ability to adapt to various microenvironments and provides a mechanism of chemoresistance. It was also observed that repression of OXPHOS was not mandatory to promote cell growth [18]. Warburg’s hypothesis proposed that cancer cells have dysfunctional or defective mitochondria, and thus, they shift to aerobic glycolysis [19]. With the discovery of the concept of plasticity, this aspect of the Warburg hypothesis is challenged. There have been several instances of upregulated OXPHOS observed in multiple forms of cancer, such as melanoma [20], pancreatic ductal adenocarcinoma (PDAC) [21], leukemia, and subset of lymphomas [22].

**Figure 3:**
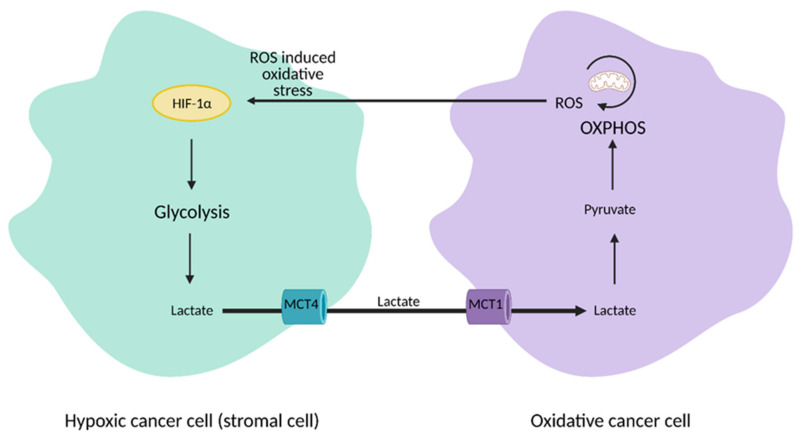
Reverse Warburg Effect. In the reverse Warburg Effect, substrates from different populations of cancer cells can be shared between each other and utilized. Oxidative cancer cells can take up lactate from hypoxic cancer cells that perform aerobic glycolysis to fuel oxidative phosphorylation (OXPHOS). Hypoxic cancer cells can also take up reactive oxygen species (ROS) from oxidative cancer cells to induce hypoxia-inducible factor 1α (HIF-1α) activation and aerobic glycolysis. This affords cancer cells an additional mechanism that enhances proliferation and survival. MCT1; monocarboxylate transporter 1, MCT4; monocarboxylate transporter 4. Figure created with BioRender.com (accessed on 26 March 2021).

The discovery of heterogeneity in tumors led to a paradigm shift from the Warburg Effect to the “reverse Warburg Effect”, where aerobic glycolysis in cancer cells metabolically supports adjacent cancer cells (Figure 3). This allows for the transfer of metabolites, such as lactate, to these cells to encourage ATP production, growth, and proliferation via oxidative phosphorylation [23,24]. This mechanism emphasizes the importance of interplay and molecular signaling in cancer cell metabolism and demonstrates that upregulation of aerobic glycolysis is not a hard and steadfast rule in the tumor microenvironment.

## 3. Glutamine Metabolism

A second key source of energy for cancer cells is the essential amino acid glutamine (Gln). Glutamine is the most consumed amino acid in cancer and the dependence on glutamine for growth is a hallmark of the disease [25]. Cancer cells deprived of glutamine rapidly die off [26], and increased glutamine metabolism in cancer is often referred to as “glutamine addiction”. Glutamine is crucial for cell survival because it plays a role in signal transduction pathways [27,28], is used as the building block for proteins, lipids, and nucleotides, and is used to synthesize glutamate (Glu), which is then converted to α-ketoglutarate (α-KG) and fed into the TCA cycle [29]. Glutamine is also used to synthesize glutathione (GSH), an important antioxidant molecule [30]. Cancer cells respond to metabolic circuit changes by increasing oxidative damage levels, which are regulated by Glu and GSH ratios in the mitochondrial membrane [31]. 

To attenuate oxidative damage and produce additional macromolecules, cancer cells increase the process of glutaminolysis, although the levels vary with heterogeneity of tumor, patient, and cancer type [32]. Glutaminolysis is the breakdown of Gln to Glu to drive the production of energy via lactate [33]. This process provides cancer cells the materials and building blocks they need for rapid growth and proliferation by avoiding OXPHOS and the generation of reactive oxygen species (ROS). 

**Figure 4:**
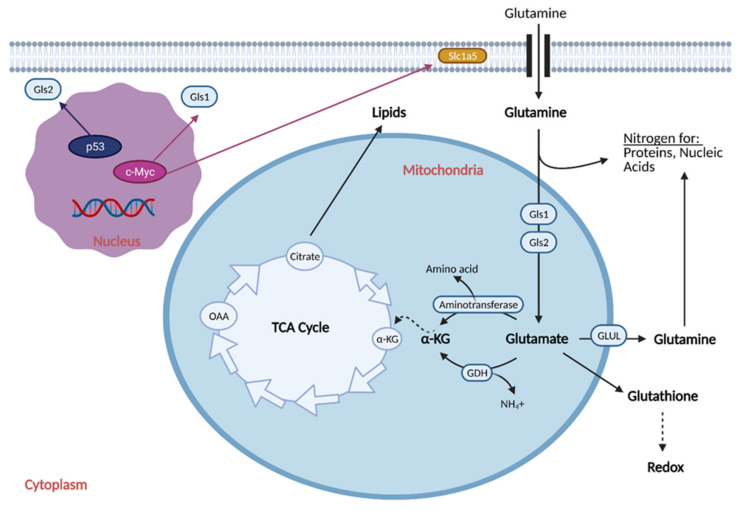
Glutamine Metabolic Reprogramming in Cancer. Cancer cells exhibit increased glutaminolysis, which is the conversion of glutamine to glutamate. This occurs as a result of upregulation of an isoform of glutaminase (Gls1) and glutamine transporter, Slc1a5, by oncogenic c-Myc. Increased glutamine uptake provides nitrogen for proteins and nucleic acids, while increased glutaminolysis provides α-ketoglutarate (α-KG) for the citric acid (TCA) cycle, resulting in increased production of lipids. Increased glutamine uptake also results in the production of glutathione, which regulates redox and helps the cell attenuate oxidative damage. Cancer cells typically exhibit downregulation of a second isoform of glutaminase, Gls2, as induction of its expression by p53 generally leads to tumor suppression. GDH; glutamate dehydrogenase, GLUL; glutamine synthetase, NH4+; ammonium, OAA; oxaloacetate. Figure created with BioRender.com (accessed on 26 March 2021).

Reprogramming the cell to perform glutaminolysis is achieved through various oncogenes and is thought to be coordinated with the reprogramming of glucose metabolism (Figure 4) [34]. One major regulator is the oncogene Myc. Myc can directly stimulate glutamine metabolism by binding to promoters of glutamine metabolism genes, such as transporter Slc1a5 [35]. It can also indirectly stimulate glutamine metabolism by repressing expression of microRNA miR-23a/b, an inhibitor of one isozyme of glutaminase (Gls1) [36]. Gls1 catalyzes the conversion of Gln to Glu [29], and thus, reversal of its inhibition is crucial for increased glutaminolysis and proliferation of tumors. Another gene with major reprogramming effects on glutamine metabolism is p53. Tumor suppressor p53 induces the expression of glutaminase isozyme Gls2 [37]. Gls2 induces OXPHOS and glutaminolysis and generally has tumor suppressor effects [31], which are opposite to that of Gls1. Other genes implicated in playing a role in reprogramming glutamine metabolism include IDH1/2 [38], glutamate dehydrogenase (GDH) [39], pyruvate carboxylase (PC) [40], phosphatidylinositide 3-kinase (PI3K) [41], signal transducer and activator of transcription 1 (STAT1) [42,43], extracellular signal-regulated kinases (ERKs) [44], and KRAS [45].

Recently, research has described a shift in glutamine nitrogen metabolism, referred to as a “second Warburg-like effect” [46]. This effect describes a change in metabolism in cancer cells from glutaminolysis to de novo nucleotide biosynthesis. Although glutaminolysis has long been considered to be a tumor promoting factor, recent evidence has demonstrated that glutaminolysis may restrict nucleotide biosynthesis and impair cancer cell proliferation. Cancer cells coordinate a shift from glutaminolysis to de novo nucleotide biosynthesis via metabolic reprogramming coordinated by GLS1 and phosphoribosyl pyrophosphate amidotransferase (PPAT), the enzyme that initiates the rate-limiting step in de novo purine nucleotide biosynthesis [47]. It has been hypothesized that the promotion of ATP generation through glutaminolysis is not an advantage for cancer cells, and thus, why they shift their glutamine nitrogen metabolism. The cancer cells can then compensate for the loss of glutamine-derived carbon sources from this metabolic shift with glucose-derived carbon sources [46]. This Warburg-like effect on glutamine metabolism may turn out to be as important as the original Warburg Effect on carbon metabolism, although more evidence is needed.

## 4. Pentose Phosphate Pathway

**Figure 5:**
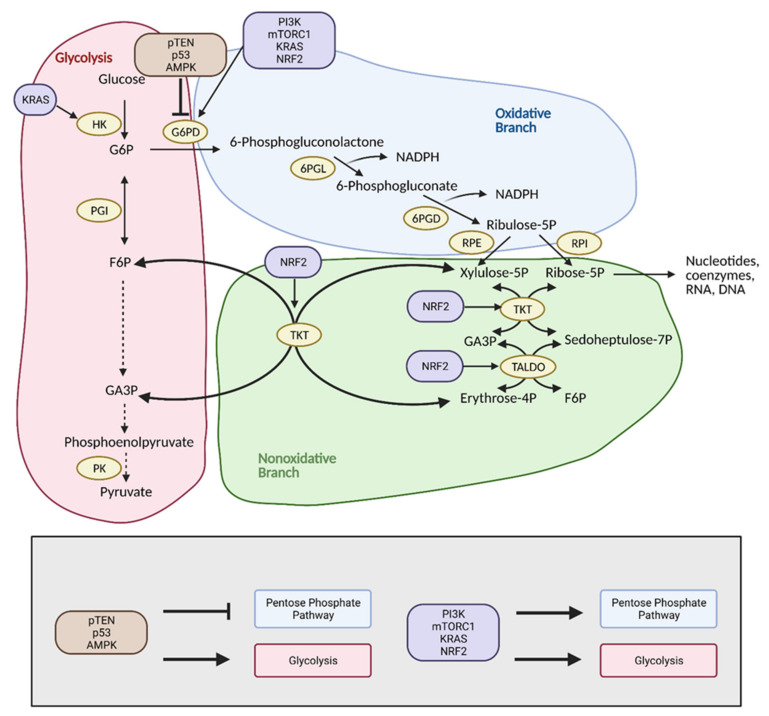
Reprogramming of the pentose phosphate pathway (PPP) in cancer. Upregulation of the PPP is achieved primarily through upregulation of glucose-6-phosphate dehydrogenase (G6PD) by PI3K, mTORC1, KRAS, and NRF2. This shunts glucose into the oxidative branch of the PPP instead of into glycolysis. Inhibitors of G6PD, pTEN, p53, and AMPK are often found mutated in cancer. Upregulation of the nonoxidative branch of the PPP in cancer occurs via NRF2, which increases transketolase (TKT) and transaldolase (TALDO) expression. There is also crosstalk between glycolysis and the PPP via KRAS, which increases hexokinase (HK) expression to upregulate glycolytic intermediates for progression into the PPP. Modulation of the PPP in this manner results in the production of energy and substrates necessary for tumor growth. F6P; fructose 6-phosphate, G3P; glyceraldehyde 3-phosphate, G6P; glucose 6-phosphate, NADPH; nicotinamide adenine dinucleotide phosphate, 6PGD; 6-phosphogluconate dehydrogenase, 6PGL; 6-phosphogluconolactonase, PK; pyruvate kinase, PGI; phosphoglucoisomerase, RPE; ribulose 5-phosphate 3-epimerase, RPI; ribose-5-phosphate isomerase. Figure created with BioRender.com (accessed on 26 March 2021).

The pentose phosphate pathway (PPP), also referred to as the hexose monophosphate shunt, is an offshoot pathway of glycolysis that plays an important role in glucose metabolism, and consequently, cancer metabolism [48]. Although focus has been heavily placed on increased glycolytic flux in cancer, recent research shows that cancer cells may metabolically reprogram themselves to direct glucose flux into the PPP [49]. After glucose is converted into glucose-6-phosphate (G6P) by hexokinase (HK), it can be further metabolized by glycolysis. Alternatively, G6P can enter the PPP, which serves to generate NADPH and precursors to both lipids and nucleotides. These molecules encourage tumor growth by providing cells with the energy and substrates necessary for the synthesis of macromolecules [49]. With the production of NADPH, the PPP also provides increased antioxidant defense for cancer cells in stress conditions to ensure their survival and proliferation, and thus, the reason why tumors tend to exhibit increased flux into this pathway (Figure 5) [50]. 

The PPP is composed of two phases or branches that undergo reprogramming in cancer through various mechanisms. Commitment of a cell to the PPP is regulated by the first phase of the PPP, the oxidative branch [51]. The first step of the oxidative branch is irreversible and is the rate-limiting step of the pathway [52]. It involves the dehydrogenation of G6P via G6P dehydrogenase (G6PD) to produce NADPH [53]. G6PD is expressed at higher rates in cancer cells, which is indicative of greater PPP flux [54]. Some specific types of cancers with notably high G6PD expression include ovarian, lung, renal, and oral cancers [55,56,57,58]. The other NADPH generating enzyme of the oxidative branch, 6-phosphogluconate dehydrogenase (6PGD), is also thought to modulate PPP flux in cancer cells in a similar manner to G6PD [49]. 

Reprogramming of the oxidative branch is primarily achieved through mechanisms involving G6PD, as this enzyme is the “gateway” between glycolysis and the PPP. Hence, a large amount of research has focused on study of regulation of this enzyme [52]. PPP oxidative branch reprogramming through G6PD is mediated by various oncogenes and tumor suppressors, including PTEN, p53, AMPK, PI3K, mTORC1, and KRAS, and molecules, such as cyclic adenosine monophosphate (cAMP), TAp73, and HSP27 [49]. PTEN, p53, and AMPK act as inhibitors of the PPP. Mutations in the genes that encode these proteins, therefore, results in increased glycolytic and PPP flux by modulating G6PD levels. Most significantly, p53 directly binds to G6PD and suppresses its enzymatic activity. When p53 is mutated or loses its function in cancer, G6PD is no longer inhibited and is free to carry out the rate-limiting step of the PPP [59]. On the other hand, PI3K, mTORC1, and KRAS, when activated, positively regulate the PPP by upregulating G6PD levels [49]. G6PD is also affected by NADP+ levels, which tend to increase during cancer as a result of higher ROS levels and oxidative stress. Increased NADP+ activates G6PD and increases PPP flux. This leads to the generation of NADPH and protection of cancer cells from DNA damage [49]. Recently, it was reported that a paralog of G6PD, hexose-6-phosphate dehydrogenase (H6PD), that is present in the endoplasmic reticulum of cells, affects PPP flux in breast and lung cancer in a similar manner to G6PD [60]. This finding may implicate the future focus of PPP cancer research.

The second branch of the PPP is the nonoxidative branch and utilizes the product of the oxidative branch, ribulose-5-phosphate (R5P), to generate glycolytic intermediates and nucleotide precursors [48]. This branch is reversible and catalyzed by the enzymes transketolase (TKT) and transaldolase (TALDO). The reversible nature of the nonoxidative branch allows for cells to adapt metabolic flux through the PPP as needed. In rapidly dividing cancer cells, the PPP is tailored to generate pentose phosphates from G6P in the oxidative branch and fructose-6-phosphate (F6P) and glyceraldehyde-3-phosphate (G3P) in the nonoxidative branch. Cancer cells increase their expression of TKT and TALDO to accelerate the nonoxidative branch of the PPP [49], with elevated levels of TKT reported in lung, breast, and prostate cancer cells [52,61,62,63], and elevated levels of TALDO reported in gastric adenocarcinoma [64]. Both TKT and TALDO expression is increased in response to Nuclear Factor, Erythroid 2-Like 2 (NRF2) activation, an important sensor of oxidative stress [65,66]. TKT expression is also stimulated by fructose, which is preferred over glucose as a substrate for nucleic acid generation in the nonoxidative branch [67].

Additionally, there is crosstalk between glycolysis and the PPP via the nonoxidative branch, which can greatly impact the regulation of these pathways in regard to cancer. The ability of the nonoxidative branch to adjust flux depending on metabolic needs allows it to act as a “bridge” between phase one of glycolysis and phase one of the PPP and allows cancer cells to exhibit “metabolic plasticity”. Specifically, the different modes of the PPP influence glycolytic flux and vice versa. Increased glycolysis upregulates intermediates, such as F6P and G3P, which can be used to generate ribonucleotides in the nonoxidative PPP. Inactivation of glycolytic enzymes, such as PFK1, can occur during oxidative stress to increase production of NADPH via the diversion of G6P to the oxidative PPP [48,54]. Crosstalk between glycolysis and the PPP allows cancer cells to reprogram their metabolism to ensure survival and proliferation.

## 5. Lipid Metabolism

**Figure 6:**
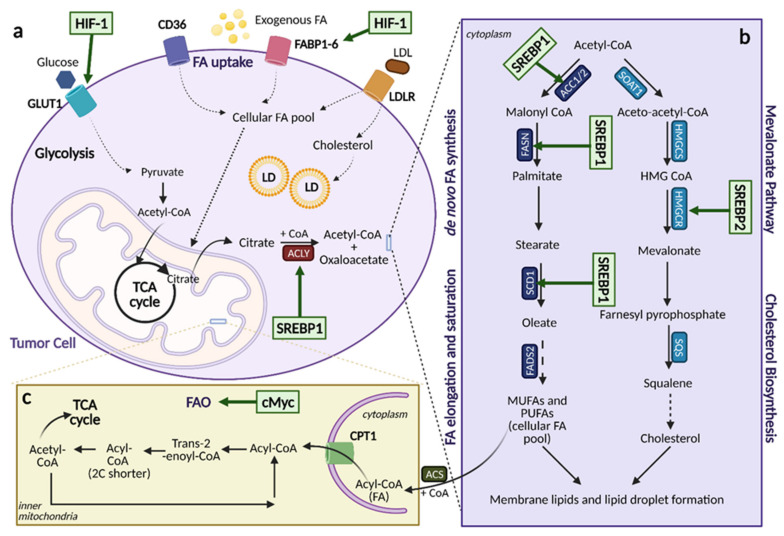
Lipid metabolic reprogramming in Cancer. An overview of lipid metabolic pathways and how they are modified in cancer. (**a**). Tumor cells take up fatty acids (FAs) using multiple trans-porters, including CD36, FA binding proteins 1-6 (FABP1-6), and a low-density lipoprotein receptor (LDLR) for low-density lipoproteins (LDL). These free FAs then become a part of the cellular FA pool where they can enter the citric acid (TCA) cycle and contribute to lipid formation. The upregulation of FA uptake in cancer occurs through hypoxia-inducible factor (HIF-1)-induced FABP1-6 over-expression. (**b**). The upregulation of lipogenesis and cholesterol biosynthesis is achieved through sterol regulatory element binding protein (SREBP) activation. SREBP1 activation induces the ex-pression of lipogenesis genes, while SREBP2 activation induces the expression of cholesterol bio-synthesis genes. (**c**). Fatty acid oxidation (FAO) can be upregulated by cMyc, depending on the cancer type as a means to counteract oxidative stress. ACC1/2: acetyl-CoA carboxylase 1/2, ACLY: ATP citrate lyase, ACS: acyl-CoA synthetase, α-KG: alpha-ketoglutarate, CoA: coenzyme A, CPT1: carnitine palmitoyltransferase 1, FADS: FA desaturases, FASN: fatty acid synthase, FPP: farne-syl-pyrophosphate, GLUT1: glucose transporter 1, HMG-CoA: hydroxy-methylglutaryl-CoA, HMGCS: hydroxy-methylglutaryl-CoA synthase, HMGCR: hydroxy-methylglutaryl-CoA reduc-tase, LD: lipid droplets, MUFA: monounsaturated fatty acids, PUFA: polyunsaturated fatty acids, SCD1: stearoyl-CoA desaturase 1, SOAT: sterol O-acyltransferase. The figure is created with Bio-Render.com (accessed on 26 March 2021). This figure is modified from Figure 1 in [78].

Aberrant lipid metabolism is one of the most pronounced metabolic alterations in cancer, and it greatly contributes to cancer cell growth and tumorigenesis [68]. Lipids, including sterols, mono/di/triglycerides, phospholipids, and glycolipids, are indispensable to cells. They serve as energy sources, as components of biological membranes, and as signaling molecules [69]. The many roles of lipids are a testament to the importance of processes that regulate their levels in cancer. Several aspects of lipid metabolism are reprogrammed in cancer, including the biosynthesis and oxidation of fatty acids (FAs), the uptake of FAs from the environment, and modification of FAs and release from other molecules (Figure 6) [68], of which the mechanisms will be discussed.

### 5.1. Lipid Acquisition: De Novo Lipogenesis and Lipid Uptake

Cells can acquire lipids in one of two ways, de novo synthesis or uptake [70]. Most lipids are derived from FAs, which are molecules containing long hydrocarbon chains. Adult cells normally obtain FAs from external sources, such as the diet or from lipids synthesized by the liver [68]. Cancer cells, however, reactivate de novo lipogenesis which removes their reliance on externally derived lipids and allows them to proliferate at a faster rate [69]. FA synthesis occurs using cytoplasmic acetyl-CoA that is generated from acetate, glucose, or glutamine. This acetyl-CoA is converted to malonyl-CoA and then 16-carbon saturated FA palmitate using the enzymes acetyl-CoA carboxylases (ACC1/2) and fatty acid synthase (FASN), respectively (Figure 6a). Palmitate can then be elongated to other FAs or desaturated using FA elongases and FA desaturases to form the cellular pool of non-essential FAs that are then further converted to form other important lipids, such as cholesterol, eicosanoids, and prostaglandins [71]. Increased FA de novo synthesis in cancer has been widely observed and this increase is essential for cancer cell growth [70,72,73,74,75,76].

**Figure 7:**
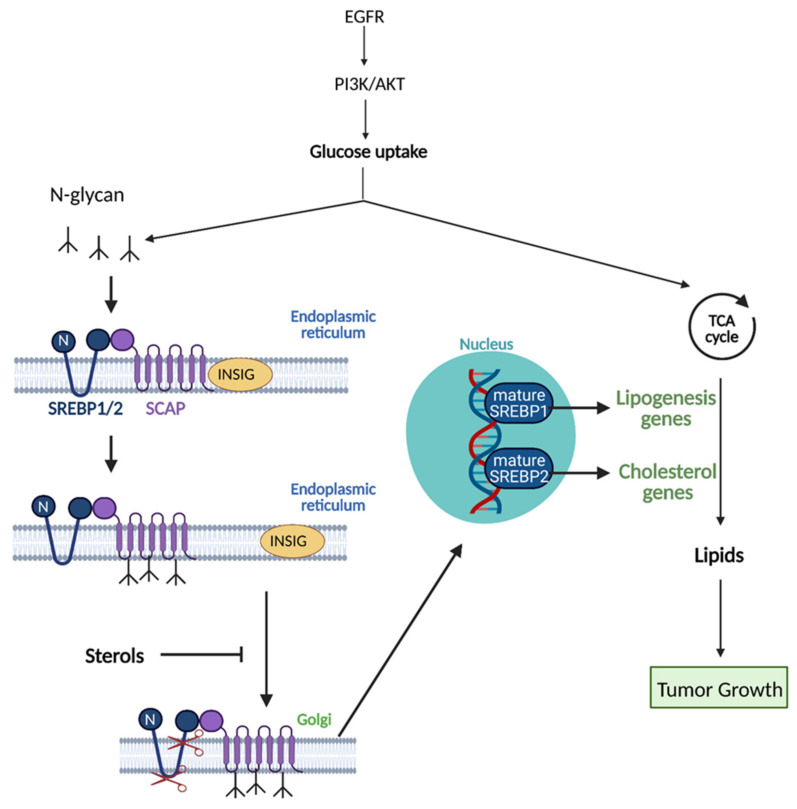
Activation of sterol regulatory element binding proteins (SREBPs) in cancer. SREBPs are the main transcription factors that regulate expression of genes involved in lipogenesis that are translated as inactive precursors in the endoplasmic reticulum associated with SREBP cleavage activating protein (SCAP) and insulin induced gene protein (INSIG). PI3K/AKT and glucose uptake results in the N-glycosylation of SREBPs, which separates the complex from INSIG and allows it to translocate to the Golgi and become proteolytically activated. Mature SREBPs bind to genes in the nucleus to induce their transcription. Mature SREBP1 preferentially binds genes involved in fatty acid (FA) synthesis while mature SREBP2 preferentially binds genes involved in cholesterol biosynthesis. The upregulation of these genes results in tumor growth. High concentrations of sterols inhibit SREBP activation. EGFR; epidermal growth factor receptor, TCA cycle; the citric acid cycle. Figure created with BioRender.com (accessed on 26 March 2021).

Cancer cells activate de novo lipogenesis by upregulating several enzymes involved in the pathway, specifically acetyl-CoA carboxylase (ACC), fatty acid synthase (FASN), and stearoyl-CoA desaturase 1 (SCD1) [70]. These enzymes are upregulated through the activation of Sterol regulatory element-binding proteins (SREBPs), which are key transcription factors involved in lipid metabolism. SREBPs are initially translated as inactive precursors in the endoplasmic reticulum and associate with the chaperone SREBP cleavage activating protein (SCAP) [71]. Glucose uptake and low sterol concentration facilitates glucose-mediated N-glycosylation of SCAP, which allows it to transport SREBPs to the Golgi where they can become proteolytically activated and bind to the promoters of effector genes in the nucleus (Figure 7) [77]. SREBP isoform SREBP-1 preferentially binds to genes involved in FA synthesis to promote their expression. SREBP activation is also regulated by upstream oncogenic signaling pathways, most predominantly by the PI3K/Akt/mTORC1 signaling axis. This axis increases the expression of enzymes needed for FA synthesis and activates ATP-citrate lyase (ACLY), which catalyzes acetyl-CoA production from citrate, which can enter into de novo lipogenesis. It also increases the production of NADPH via the activation of NRF2, which is used as a cofactor in FA synthesis reactions [68,69].

Regardless of the signaling molecules involved, increased *de novo* lipogenesis provides cancer cells with the ability to shunt into different biosynthetic pathways to create lipids with a wide variety of functions that allow them to adapt and respond to their surroundings and ensure continued proliferation. Specifically, increased FA synthesis reduces the number of polyunsaturated FAs (PUFAs) and increases the number of monounsaturated FAs (MUFAs). This helps provide protection from lipid peroxidation as PUFAs are subject to peroxidation in the presence of ROS. Increased FA synthesis in cancer cells also confers protection from ROS, contributes to pro-angiogenic signaling, and provides an escape from immune surveillance [79]. 

Besides de novo lipogenesis, cancer cells also acquire a diverse pool of lipids by increasing lipid uptake [69]. Lipid uptake can occur via multiple routes, including the use of specialized transporters such as CD36 fatty acid translocase or the fatty acid transport proteins (FATPs of the SLC27 family of solute carriers), or receptor-mediated endocytosis of low-density lipoprotein (LDL) particles via the LDL receptor (LDLR), all of which are highly expressed in various types of cancer (Figure 6a) [68]. The uptake of exogenous FAs also promotes migration and metastasis. Through the remodeling of cellular FA composition, cancer cells can facilitate changes in membrane fluidity that promote cell migration and cancer progression [79,80]. Additionally, the uptake of lipids from the environment allows tumors to maintain their lipid pool, even in times of stress. For example, under hypoxic conditions, the conversion of saturated FAs into monounsaturated FAs is hindered, as the enzyme catalyzing the reaction, stearoyl-CoA desaturase-1 (SCD-1), requires oxygen. Hypoxic cells can compensate by taking up exogenous lysophospholipids to survive. Exogenous FA uptake is mediated by the master regulator, HIF-1α, and its control of overexpression of lipid-binding proteins, such as FA-binding protein 4 (FABP4) [69].

### 5.2. Lipid Storage and Export

One consequence of increased de novo lipid synthesis and uptake is that, with an excess of lipids, cancer cells must store them. Excess lipids are stored as lipid droplets, which are produced via conversion of cellular lipids to triglycerides and cholesteryl esters in the endoplasmic reticulum by sterol O-acyltransferase 1 (SOAT1), also known as acyl-CoA acyltransferase 1 (ACAT1) [70]. Cancer cells exhibit an increased number of lipid droplets compared to normal cells. These lipid droplets help maintain lipid homeostasis, prevent lipotoxicity, regulate autophagy, maintain ER and membrane homeostasis, and also provide a source of ATP and NADPH through their breakdown by lipophagy followed by β-oxidation in times of metabolic stress [69]. An accumulation of lipid droplets is found in several types of cancer, including breast, brain, liver, cervical, prostate, colon, skin, bile duct, clear-cell renal carcinoma, ovarian, and pancreatic cancer [81].

### 5.3. Lipolysis

Tumor cells also acquire FAs through the breakdown of lipid droplets through a process called lipolysis. Lipolysis refers to the breakdown of lipid droplets by lipoprotein lipase (LPL) to release free FAs [82]. These free FAs can then be taken up by CD36 and used to support increased growth. Increased expression of LPL occurs in breast cancer [83], non-small cell lung cancer [84], and chronic lymphocytic leukemia [85], with breast cancer also exhibiting increased CD36 expression. Increased lipolysis is associated with cachexia, a clinical manifestation of cancer referred to as “fat wasting”. Cachexia is weight loss due to muscle and adipose tissue (AT) depletion that is found in multiple types of cancer and is associated with poorer prognosis. Although efforts in the past have mainly focused on muscle loss, recent studies focus on the role of lipolysis in this process, as the loss of AT is mainly due to increased induction of lipolysis [86]. Cytokines, such as TNF-α and IL-6, and lipid mobilizing factor, zinc-α2-glycoprotein (ZAG), play a major role in the upregulation of lipolysis in cancer, although additional research is needed to understand the mechanism underlying this change [86].

### 5.4. Fatty Acid Oxidation

The process of lipolysis breaks down lipid droplets to free FAs and these free FAs can then be further broken down by fatty acid oxidation (FAO), referred to as β-oxidation. While the role of FA synthesis in cancer has been widely established, the role of β-oxidation has not been as well defined and is a newer area of study cancer metabolism. As a source of ATP and NADPH, β-oxidation provides the energy and reducing power for biosynthesis and a means to counteract oxidative stress. Most research, however, has focused on the generation of ATP through the Warburg Effect. NADPH can be produced via other metabolic pathways, such as the PPP, suggesting that FAO does not play a major role in the oncogenic landscape. Furthermore, malonyl-CoA, an intermediate of lipogenesis, coordinates the activity of both lipogenesis and FAO. Malonyl CoA acts as an inhibitor of the FAO rate-limiting enzyme carnitine palmitoyltransferase 1 (CPT1), supporting the idea that FA synthesis and FAO cannot occur at the same time. However, new evidence suggests that FAO may play a greater role in cancer growth and metastasis than previously thought [87].

Recent studies have demonstrated that there is increased expression of several FAO enzymes in cancer, including CD-36, CPT1 isoforms A, B, and C, carnitine transporter CT2 [42], and Acyl-CoA synthetase long chain 3 [83,87]. Consistent with this observation, several types of cancer exhibit increased FAO, such as triple negative breast cancer (TNBC) [88], gastric cancer [89], glioma [90], and prostate cancer [91]. These types of cancer rely on FAO as a main source of ATP for rapid growth and even prefer to metastasize to tissues rich in adipocytes [87]. Non-glycolytic tumors, such as those in prostate cancer, employ FAO as their main bioenergetic pathway [91]. Increased expression of FAO enzymes and upregulation is achieved by overexpression of oncogenic c-Myc (Figure 6c) [87]. As a generator of NADPH, FAO also helps cancer cells respond to oxidative stress and avoid cell death [92]. Additionally, FAO has been implicated in metastasis through its potential role in the reprogramming of cancer stem cells [93]. Taken together, the data suggest that FAO plays an important role in cancer metabolism.

### 5.5. Mevalonate Pathway

The generation of important lipids, such as cholesterol, vitamin D, and lipoproteins, through reprogramming of the mevalonate pathway (MVA) in cancer has been extensively studied, with a focus placed on cholesterol biosynthesis. The MVA uses acetyl-CoA derived from glycolysis to generate its products, with mevalonate production catalyzed by 3-hydroxy-3-methylglutaryl-CoA reductase (scdCR) being the rate-limiting step of the entire pathway. Mevalonate is then converted to isopentenyl pyrophosphate (IPP) and later, farnesyl pyrophosphate (FPP). FPP is critical for production of squalene, the precursor to cholesterol. Cholesterol itself is an important component of cell membranes and is the precursor to hormones, bile acids, and lipid rafts [94].

Many enzymes of the MVA are often overexpressed in cancer, including HMGCR, farnesyl diphosphate synthase (FDPS), geranylgeranyl pyrophosphate synthase (GGPPS), squalene synthase, and squalene epoxidase [94]. The transcription of these enzymes is controlled by SREBPs, in manner similar to de novo lipogenesis, with isoform SREBP2 showing a preference for the promoters of MVA and cholesterol biosynthesis genes (Figure 6b) [68]. Again, like de novo lipogenesis, SREBP2 is mediated by the PI3K/Akt/mTORC1 signaling axis. This results in increased HMGCR expression, and thus, increased flux through the MVA. SREBP2 can also interact with mutant p53 to drive the post-translational modification of oncogenes, such as the farnesylation of Ras, and regulates mediators of epigenetic changes, such as histone deacetylases (HDACs) and DNA methyltransferases (DNMTs) [69,95]. Increased expression of HMGCR in cancer leads to increased production of cholesterol, which provides a continuous resource for membrane synthesis in dividing cells and of estrogen and androgens to support tumorigenesis [94]. As a result, inhibition of cholesterol biosynthesis with statins greatly impairs cancer growth [96,97]. 

In addition to cholesterol, other products of the MVA pathway play roles in cancer cell growth. One such product is ubiquinone, a key electron transfer molecule in respiration. Oxidative phosphorylation is an active metabolic pathway in many tumors, and hence, ubiquinone is an important product of the MVA for continued cell proliferation. Ubiquinone is also a regulator of ROS, and more recently, it was reported that ubiquinone supports pyrimidine biosynthesis in colorectal and pancreatic cancer [69]. Thus, the MVA pathway contribute a number of molecules needed for cancer cell survival.

## 6. The Tricarboxylic Acid (TCA) Cycle

The tricarboxylic acid (TCA), or Krebs cycle, is a central hub of metabolism that takes place in the mitochondrial matrix and has the primary task of providing NADH and flavin adenine dinucleotide (FADH2) to be reduced in OXPHOS for ATP production [98]. It is also a source of intermediates, such as citrate, oxaloacetate, and succinyl-coenzyme A, that can be used as building blocks for the synthesis of lipids, aspartate, and other key macromolecules [99]. It was previously thought that cancer cells bypass the TCA cycle and favor aerobic glycolysis. Recent evidence, however, suggests that cancer cells do rely heavily on the TCA cycle for energy production and growth [100]. This is achieved through the uncoupling of glycolysis from the TCA cycle, which allows for the use of alternate fuel sources to support increased metabolic demands [100]. 

**Figure 8:**
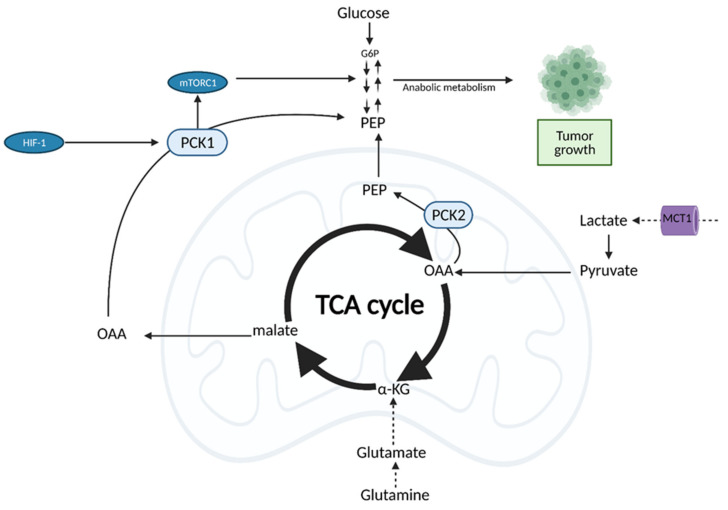
Oncogenic Regulation of the citric acid (TCA) cycle to support tumor growth. Unlike normal cells which primarily utilize glucose for input into the TCA cycle, cancer cells rely on alternative substrates, such as glutamate produced via glutaminolysis and lactate. TCA cycle flux is modulated by phosphoenolpyruvate carboxykinase (PEPCK), which has both cytosolic and mitochondrial isoforms (PCK1/2). PEPCK is overexpressed via hypoxia-inducible factor (HIF-1) and preferentially uses OAA derived from lactate as a substrate. Increased anaplerosis into the TCA cycle is compensated by the cataplerotic conversion of oxaloacetate (OAA) to phosphoenolpyruvate (PEP) via PEPCK. Overexpression of PEPCK promotes cancer cell growth via a truncated form of gluconeogenesis to glycolytic intermediates. These intermediates can be used for anabolic metabolism to support tumor growth. α-KG; alpha-ketoglutarate, G6P; glucose 6-phosphate, MCT1; monocarboxylate transporter 1. Figure created with BioRender.com (accessed on 26 March 2021).

Both normal cells and tumor cells can catabolize every type of fuel that feeds the TCA cycle, including glucose, glutamine, and fatty acids; however, they differ in the rate of utilization and uptake of each fuel. While normal cells primarily use the conversion of glucose to pyruvate to fuel the TCA cycle, cancer cells typically shunt glucose away from the TCA cycle for breakdown in aerobic glycolysis. As a result, cancer cells are more dependent on glutamine and fatty acids to fuel the TCA cycle (Figure 8), although the exact levels of substrate utilization vary based on cancer type [100]. The metabolism of both glutamine and fatty acids is reprogrammed in cancer cells. This metabolic reprogramming allows for increased utilization of these alternate fuel sources and, thus, continuation of flux through the TCA cycle to support growth. TCA cycle flux is modulated by phosphoenolpyruvate carboxykinase (PEPCK), a key enzyme in gluconeogenesis often overexpressed in cancer [101]. Upregulation of the expression of either the cytosolic isoform, PCK1, or the mitochondrial isoform, PCK2, is dependent on cancer type. PEPCK expression is regulated by HIF-1 and promotes cancer cell growth via the cataplerotic conversion of oxaloacetate (OAA) to phosphoenolpyruvate (PEP), and subsequently pyruvate, in a truncated form of gluconeogenesis. The increased cataplerosis results in increased flux through the TCA cycle in nutrient starvation conditions seen in the tumor microenvironment, and therefore, must be compensated for with increased anaplerosis into the TCA cycle. This is accomplished via glutamine and glutaminolysis, which allows for utilization of non-carbohydrate sources for anabolic reactions that create lipids and nucleotides anaplerosis to be shuttled into the TCA cycle [101,102].

Importantly, cancer cells can also use other substrates for the TCA cycle, such as lactate. Although lactate was primarily considered a byproduct of aerobic glycolysis in the tumor environment, recent studies show that pancreatic, breast, and lung cancers utilize lactate for the TCA cycle, and even preferentially, over glucose [103,104,105]. Lactate utilization is also modulated by PEPCK, and specifically the mitochondrial isoform, PCK2. PCK2 preferentially converts OAA derived from lactate, allowing it to be used in anabolic reactions in times of glucose deprivation [106]. The “metabolic plasticity” of cancer cells in their use of lactate and other substrates to continue TCA cycle flux is yet another mechanism they employ to promote rapid growth. 

There are also several mutations in TCA cycle enzymes associated with tumor proliferation, including mutations in aconitase (ACO2), citrate synthase (CS), succinate dehydrogenase (SDH), fumarate hydratase (FH), and isocitrate dehydrogenase (IDH) [105]. Notably, SDH and IDH mutations lead to increased production of ROS and promote tumorigenesis while FH mutations lead to the accumulation of fumarate, which can act as oncometabolite and allows for HIF stabilization [105,106,107].

## 7. Acetate

In addition to metabolizing glucose, glutamine, and lipids, some cancer cells are also capable of metabolizing exogeneous acetate to facilitate growth. Acetate, when ligated to coenzyme A (acetyl CoA), plays a role in a number of regulatory and biosynthetic processes, such as post-translational modification and the synthesis of fatty acids, nucleotides, and amino acids [108]. Before cancer cells can convert acetate to acetyl CoA, acetate must be produced from microbial fermentation, alcohol oxidation, or obtained from the diet and then taken up into cells using transporters. After cellular uptake, acetate can be converted to acetyl CoA by mitochondrial-localized acetyl-CoA synthetase 1 (ACSS1) and nucleocytosol-localized ACSS2 where it can then contribute to fatty acid synthesis, the TCA cycle, and histone acetylation [109]. ACSS2 and its role in cancer has been extensively studied, with expression levels found to correlate with tumor aggressiveness. This indicates that certain cancer cells may have an “acetate addiction” similar to the well-studied “glutamine addiction”, as the proliferation of normal cells is not affected by a lack of ACSS2 [108]. 

**Figure 9:**
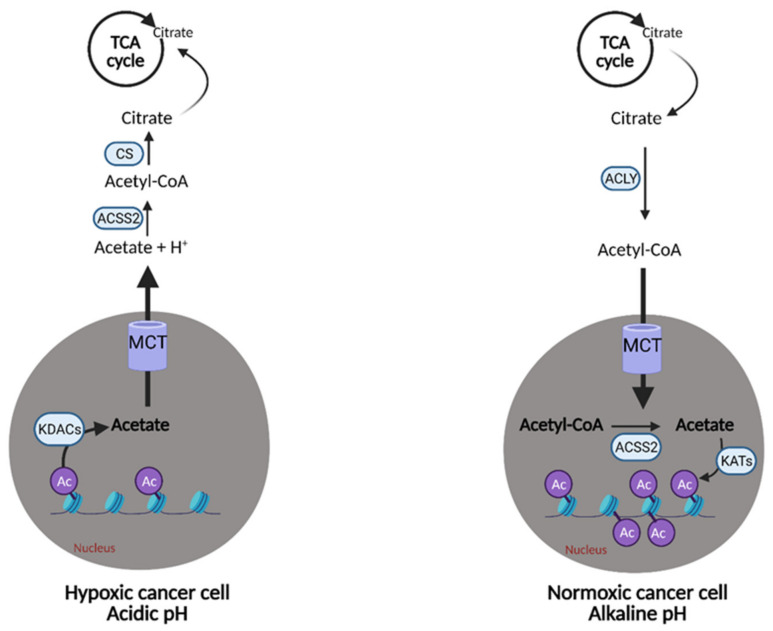
Metabolic plasticity of acetate in cancer. Cancer cells can alter the concentration of free acetate based on conditions in the tumor microenvironment. In hypoxic/acidic conditions, when oxidative phosphorylation (OXPHOS) is compromised or when there is low availability of exogenous fatty acids (FAs), cancer cells can release free acetate to raise the pH inside of the cell or convert it into acetyl-CoA for use in the citric acid (TCA) cycle. This is accomplished via the release of acetate from acetylated histones in the nucleus by lysine deacetylases (KDACs). Alternatively, cancer cells with functional OXPHOS or an excess of free FAs, can uptake free acetate via the acetylation of histones in the nucleus by lysine acetyltransferases (KATs). This buffering system provides cancer cells with another survival and growth mechanism. Ac; acetyl group, Acetyl-CoA; acetyl-coenzyme A, ACLY; adenosine triphosphate (ATP) citrate lyase, ACSS2; acetyl-CoA synthetase 2, CS; citrate synthase, H+; hydrogen, MCT; monocarboxylate transporter. Figure created with BioRender.com (accessed on 26 March 2021).

Acetate also represents another route of “metabolic plasticity” for cancer cells. In hypoxic conditions, when OXPHOS is compromised, or when the availability of exogenous FAs is low, acetate can be converted to acetyl CoA for use in the TCA cycle or production of biomass. The role of acetate in histone acetylation can be exploited by cancerous cells as an adaption and growth mechanism. Lysine acetyltransferases (KATs) catalyze the transfer of the acetyl group from acetyl-CoA for acetylation, and conversely, lysine deacetylases (KDACs) containing zinc (Zn-KDACs) catalyze the deacetylation of histones, which releases free acetate. This free acetate can then be exported or converted back to acetyl CoA via acetyl CoA synthetases. Zn-KDACs are often over-expressed in various cancers, resulting in an increased release of free acetate from the cells, providing a mechanism of pH adaptation in cancer cells. In the acidic tumor microenvironment, the cell can release free acetate to buffer itself and alleviate metabolic stress for a short period of time (Figure 9), thus, supporting survival and growth until other metabolic pathways can catch up and compensate [109].

## 8. One-Carbon Metabolism

**Figure 10:**
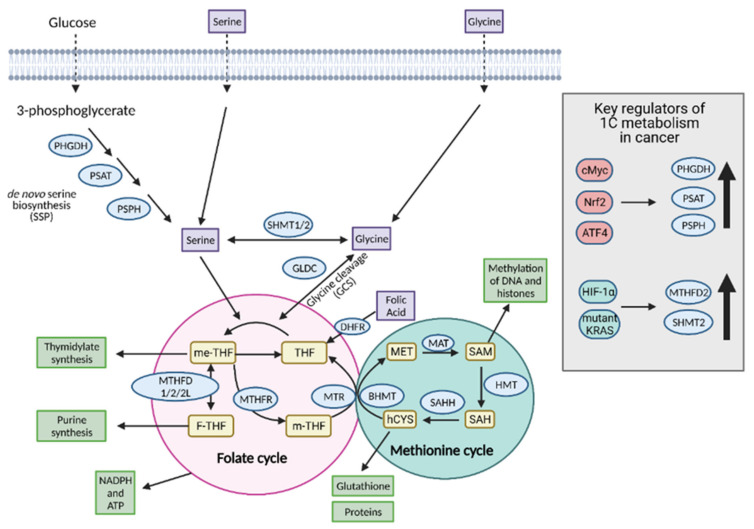
Regulation of one-carbon (1C) metabolism in cancer. Serine and glycine are transported into the cell or synthesized in the cell and serve as input molecules for 1C metabolism. Serine is synthesized from the glycolytic intermediate, 3-phosphoglycerate, via the de novo serine biosynthetic pathway (SSP) and glycine can be synthesized from serine via serine hydroxymethyltransferase 1 (SHMT1) in the cytoplasm or SHMT2 in the mitochondria. Upregulation of the SSP in cancer occurs via cMyc, Nrf2, and ATF4. Upregulation of the mitochondrial isoform SHMT2 occurs in cancer via hypoxia-inducible factor 1α (HIF-1α) and mutant KRAS-dependent pathways. Serine and glycine can both enter the early part of 1C metabolism, the folate cycle, although cancer cells tend to preferentially utilize serine over glycine. Upon entering the folate cycle, serine and glycine can donate 1C units to tetrahydrofolate (THF) to form 5,10-methylenetetrahydrofolate (me-THF). From there, one of three transformations can occur that lead to thymidylate synthesis, purine synthesis, or methionine synthesis via the coupling of the methionine synthase (MTR) reaction to conversion of homocysteine (hCYS) to form methionine (MET). The latter connects the folate cycle to the methionine cycle. The methionine cycle can be used to generate glutathione, proteins, and S-adenosylmethionine (SAM), all of which are important for cancer cell growth. ATP; adenosine tripihosphate, BHMT; betaine-homocysteine S-methyltransferase, DHFR; dihydrofolate reductase, F-THF; 10-formyltetrahydrofolate, GLDC; glycine dehydrogenase, HMT; histone methyl transferase, MAT; methionine adenosyltransferase, m-THF; 5-methyl-tetrahydrofolate, MTHFD 1/2/2L; methyltetrahydrofolate dehydrogenase 1/2/2L, MTHFR; methylenetetrahydrofolate reductase, NADPH; nicotinamide adenine dinucleotide phosphate, PHGDH; phosphoglycerate dehydrogenase, PSAT; phosphoserine aminotransferase; PSPH; phosphoserine phosphatase, SAH; S-adenosyl homocysteine, SAHH; SAH hydrolase. Figure created with BioRender.com (accessed on 26 March 2021).

One-carbon (1C) metabolism involves the transfer and cycling of 1C-groups between various acceptor groups for biosynthesis [110]. 1C metabolism controls the synthesis of purines, thymidine, glutathione, and S-adenosylmethionine (SAM), which are ultimately converted to proteins, lipids, nucleic acids, and other cofactors. It contributes to the energy balance by supplying ATP and NADPH to the cell and, therefore, it can confer “metabolic plasticity” by allowing a cell to adjust its nutrient status based on redox and epigenetic statuses [111]. For these reasons, 1C metabolism is upregulated in cancer cells and is a major player in tumor proliferation (Figure 10).

### 8.1. Input Molecules for 1C Metabolism

Non-essential amino acids (NEAAs) typically serve as the input molecules for 1C metabolism. NEAAs serine and glycine predominantly serve as the input molecules, donating their carbon groups to the latter core cycles of 1C metabolism, the folate and methionine cycles. The folate and methionine cycles can then redistribute these carbon groups to generate the key products of 1C metabolism [111]. The input molecules of 1C metabolism are interconnected and exhibit reprogramming in cancer, of which the mechanisms will be discussed.

#### 8.1.1. Serine

Serine is the major one-carbon donor and the central amino acid in 1C metabolism; thus, regulation of its bioavailability is crucial. Cells obtain serine for 1C metabolism through either exogenous uptake or de novo biosynthesis. Both increased serine biosynthesis and uptake is typical in cancer cells, [111,112] as serine is an important precursor to many molecules, including glycine, cysteine, sphingolipids, and 5,10-methylenetetrahydrofolate (me-THF), a precursor in purine synthesis [113]. Therefore, an increase in serine bioavailability is of great value to rapidly growing cancer cells. Cancer cells quickly consume exogenous serine, resulting in a sharp drop in intracellular serine levels. To continue rapid proliferation, cancer cells reprogram serine metabolism to further increase the amount of serine available, primarily through the serine biosynthetic pathway. 

The serine biosynthetic pathway (SSP) consists of 3 steps, involving the conversion of glycolytic intermediate, 3-phosphoglycerate (3-PG), to serine via reactions catalyzed by phosphoglycerate dehydrogenase (PHGDH), phosphoserine aminotransferase (PSAT1), and phosphoserine phosphatase (PSPH) [111]. Cancer cells increase serine biosynthesis by increasing the expression of these enzymes [114,115,116,117]. Significantly, PHGDH is amplified in a number of cancers, including breast cancer, melanoma, lung cancer, colon cancer, and neuroendocrine prostate cancer (NEPC), and higher PHGDH expression is associated with increased serine biosynthesis and poorer survival and prognosis [110,111,118]. Serine biosynthesis is induced in response to metabolic stress, such as glucose and glutamine depletion, and a depletion of serine itself. Serine is an activator of the M2 isoform of the glycolytic enzyme, pyruvate kinase (PKM2); therefore, serine depletion reduces flux through the last step of glycolysis and instead directs 3-PG into the SSP [110]. Serine depletion is detected by the general control non-derepressible 2 (GCN2) pathway, involving activating the transcription factor 4 (ATF4), which promotes increased expression of the SSP enzymes [97]. Furthermore, both c-Myc and Nrf-2 can induce the expression and genetic modification of SSP enzymes to increase serine biosynthesis (Figure 10) [110,118]. Interestingly, both mutant and wild type p53 are involved in supporting cancer cell proliferation under conditions of serine starvation as well, mainly by preserving cellular antioxidant capacity [119]. In addition to feeding into the folate and methionine cycles, serine biosynthesis generates an important metabolite during the transamination step, α-KG, which refuels the TCA cycle and bolsters cancer metabolism, thus, showing multiple benefits to the upregulation of serine biosynthesis in tumors [118]. Overall, coordinated induction of SSP enzymes by regulatory molecules in cancer cells helps replenish serine concentration to levels necessary for growth and proliferation.

#### 8.1.2. Glycine

Another donor of carbon groups in 1C metabolism is glycine, which aids in producing glutathione and purines, and supports proliferation and antioxidant defense. While the role of serine in cancer cell proliferation is widely accepted, the impact of glycine levels on the process remains up for debate. Glycine, like serine, can be taken up exogenously by membrane transporters, or it can be generated from serine in the cytoplasm or mitochondria. Increased glycine consumption and expression of enzymes involved in the mitochondrial glycine biosynthesis pathway, such as mitochondrial serine hydroxymethyltransferase 2 (SHMT2), methylenetetrahydrofolate dehydrogenase 2 (MTHFD2), and MTHFD1-like (MTHFD1L), are associated with higher rates of growth and proliferation in cancer cells [111]. Once synthesized, glycine can donate carbons via the glycine decarboxylase complex (GLDC), a component of the glycine cleavage system (GCS), to the folate and methionine cycles to support nucleotide biosynthesis and may even substitute for serine in some instances [118]. Overexpression of GLDC is common in many cancer types and is associated with growth and tumorigenesis [118]. 

Despite providing one-carbon units, cancer cells prefer to utilize serine over glycine. Moreover, high levels of glycine can inhibit cancer cell growth by preventing the conversion of glycine into purines and instead driving the conversion of glycine into serine [111,120]. Serine is converted to glycine and me-THF by SHMT1 in the cytoplasm and SHMT2 in the mitochondria, respectively. In contrast, an excess of glycine can drive the reverse reaction at the expense of reducing the pool of me-THF, which is needed to maintain nucleotide biosynthesis [121]. Thus, the cell prefers to remove glycine either by export out of the cell or cleavage by the GLDC to prevent the reverse reaction and maintain a high growth rate [111]. Taken together, the contrasting data suggest that while glycine biosynthesis is necessary in one-carbon metabolism, an excess of glycine can be detrimental, and therefore, glycine levels must be tightly controlled for cancer cell proliferation. However, it is likely that the contribution of glycine to 1C metabolism is dependent on tumor type and environment, and a subset of tumors may prefer to utilize glycine over serine.

#### 8.1.3. Folate and Methionine Cycles

The folate and methionine cycles are coupled together and form the core pathway of 1C metabolism. Both of these cycles integrate carbon units derived from either serine or glycine to form the molecules needed for DNA and RNA biosynthesis, and NADPH and ATP for energy and redox homeostasis. The folate cycle involves the reduction of folate, commonly known as vitamin B9, by dihydrofolate reductase (DHFR) to the biologically active tetrahydrofolate (THF) [122]. THF can then accept one-carbon units transferred by SHMT1/2 or GLDC from serine and glycine, respectively, to form me-THF [121]. After accepting the carbon, me-THF can undergo one of three transformations that alter its oxidation state to form compounds that aid in thymidylate, purine, and methionine biosynthesis, with each of the transformations closing its respective loop of the folate cycle. Methionine biosynthesis couples the folate cycle to the methionine cycle. 5-methyl-tetrahydrofolate (m-THF) formed from the folate cycle can react with homocysteine to form methionine in the methionine cycle. Methionine is then converted to SAM, the primary donor of methyl groups in a cell [110,111]. After conversion to SAM, the methionine cycle is closed by conversion back to homocysteine, which can be used to generate proteins through the transsulfuration pathway (Figure 10) [110].

Altered flux through both of these cycles is found in cancer cells. Although the folate cycle can occur in both the cytoplasm and the mitochondria, cancer cells typically overexpress mitochondrial 1C metabolism enzymes, such as MTHFD2 and SHMT2, linking mitochondrial folate metabolism to cancer progression [111,123]. MTHFD2 and SHMT2 expression are upregulated by HIF-1α and activating mutations in KRAS, respectively [110]. The mitochondrial pathway is hypothesized to be preferred because it contributes to the maintenance of mitochondrial NADH and NADPH levels, and thus, redox homeostasis. It also has the potential to contribute to ATP regeneration via MTHFD1L-mediated reaction that produces formate, which can cross the mitochondrial membrane and fuel cytosolic reactions. The upregulation of the mitochondrial folate cycle strongly correlates with the sensitivity of cancer cell lines to chemotherapy drugs, demonstrating that this cycle has the ability to affect metabolic reprogramming in a compartment-based way and that cancer cells are dependent on 1C metabolism for proliferation [124].

Altered methionine cycle flux is also crucial for driving cancer cell proliferation. Specifically, cancer cells exhibit a dependence on exogenous methionine that is known as methionine dependence or the Hoffman effect [125]. The Hoffman effect describes how cancer cells are unable to grow when methionine is replaced with its precursor, homocysteine. As these cells can still produce methionine from homocysteine, this phenomenon is most likely caused by increased demand for the metabolites generated from exogenous methionine and a need for altered metabolic flux. High methionine cycle activity causes methionine consumption to greatly exceed its regeneration, leading to an addiction to exogenous methionine [126]. The reason for preferential exogenous methionine uptake over synthesized methionine is not known. It is suggested that the key to understanding the Hoffman effect involves SAM synthesis. Cancer cells exhibiting the Hoffman effect are not limited by the availability of methionine, but by the availability of SAM, as supplementing cells exhibiting the Hoffman effect with SAM restores their proliferation. SAM affects methylation levels in a cell, and since increased proliferation rates in cancer cells require more methylation activity, cancer cells require greater amounts of SAM. When SAM levels become too low, cancer cells compensate by going into cell cycle arrest to preserve cellular integrity and epigenetic stability, referred to as the SAM-checkpoint. As cancer cells require higher levels of SAM to survive, their SAM-checkpoint is extremely sensitive to prevent cell death and maintain proliferation [125,127].

The Hoffman effect is also connected to the folate cycle and 1C metabolism. The folate cycle is especially important for regulating SAM levels because it is needed for the re-methylation of homocysteine to methionine and the production of ATP, both of which are required for SAM synthesis. The Hoffman effect also links to glycolysis and the Warburg Effect. Glycolysis is connected to the folate and methionine cycles through 3-PG, which is required for serine biosynthesis and its subsequent contribution of carbon units to the folate and methionine cycles. Therefore, the reliance of cancer cells on serine for proliferation may be linked to both glycolytic and 1C metabolism flux [125].

### 8.2. Molecules Produced as a Result of 1C Metabolism

Altered 1C metabolism in cancer results in the production of a number of important molecules, including nucleotides, glutathione, SAM, NADPH, and ATP (Figure 10) [111], all of which are necessary for the growth and proliferation of cancer cells.

#### 8.2.1. Nucleotides

The main outputs of 1C metabolism are purines and pyrimidines, which provide the building blocks for DNA synthesis. Nucleotide biosynthesis requires cofactors generated through 1C metabolism pathways [111]. In cancer, both de novo purine and pyrimidine biosynthesis are upregulated to support increased growth rates. Purine nucleotides are synthesized through a series of steps. The first of these requires R5P from the PPP, the incorporation of two one-carbon units, and one molecule of glycine to produce inosine monophosphate (IMP), the precursor to all purine nucleotides [111]. The upregulation of serine biosynthesis is necessary in cancer cells to prevent a build-up of precursors upstream of IMP prior to the input of 1C units. 1C metabolism also helps to produce pyrimidine (thymidylate) nucleotides. The methylation of deoxyuridine monophosphate (dUMP) to deoxythymine monophosphate (dTMP) requires me-THF produced from 1C metabolism as a methyl donor [121,128].

Cancer cells upregulate de novo nucleotide biosynthesis through both the aforementioned upregulation of 1C metabolic pathways and downstream pathways. A major mechanism specific to de novo nucleotide biosynthesis includes the upregulation of thymidylate synthase, inosine synthetase, and rate-limiting enzyme phosphoribosyl-pyrophosphate synthetase 2 (PRPS2) via c-Myc expression [129,130].

#### 8.2.2. SAM

SAM is the primary regulator of methylation levels in a cell that is produced from 1C metabolism. It is required for the methylation of DNA, histones, and other substrates [110]. Methylation is often altered in cancer, with tumors frequently displaying global hypermethylation and gene specific methylation. These altered patterns of methylation can affect the proliferation of cancer cells by modulating key epigenetic enzymes, for example, the suppression of tumor-suppressor gene promoters [121]. Various mechanisms control SAM synthesis and methylation patterns, such as an active mTORC1-mediated ATF4-SSP/one-carbon metabolism axis, which upregulates SAM synthesis, and serine-threonine kinase (LKB1) deletion, which increases the expression of SSP-related enzymes, thereby, increasing SAM synthesis and methylation levels. Furthermore, ATP generated from increased serine and glycine metabolism participates in the conversion of methionine to SAM [121].

#### 8.2.3. Glutathione

Glutathione is a tripeptide consisting of cysteine, glycine, and glutamate; thus, its production is regulated by 1C metabolic pathways [131]. Glutathione is the main antioxidant molecule of the cell as it maintains the NADP+/NADPH ratio and, consequently, redox balance. Cancer displays increased glutathione synthesis, as this prevents against the accumulation of dangerous ROS that would disrupt the intricate balance of antioxidant levels crucial for survival. This is achieved by increased flux through the SSP and the mitochondrial folate cycle mediated by HIF-1 [132], imparting cancer cells with survival and proliferation advantages.

#### 8.2.4. NADH/NADPH and ATP

NADH, NADPH, and ATP are important for multiple metabolic and biosynthetic pathways. As such, rapidly proliferating cancer cells display an increased need for these molecules. There are several reactions of 1C metabolism that contribute to the generation of NADH, NADPH, and ATP. This includes the production of NADPH and ATP by MTHFD1 in the folate cycle, NADH production by MTHFD2, and NADPH production by MTHFD2L [121]. Specifically, the MTHFD2 reaction runs at a higher rate than the number of one-carbon units needed for purine biosynthesis, thus, allowing the production of additional NADH that can be diverted to OXPHOS for ATP production [128]. Additionally, the reaction catalyzed by MTHFD1 and the resulting production of cytosolic NADPH can fuel fatty acid synthesis [121]. These reactions provide a source of energy generation to rapidly growing cancer cells, in addition to aerobic glycolysis and other aforementioned pathways.

## 9. Other NEAAs

In addition to NEAAs that are heavily studied, i.e., serine, glycine, glutamate, and glutamine, the NEAAs alanine, aspartate, asparagine, arginine, cysteine, and proline are emerging as players in the tumor metabolic landscape [133].

### 9.1. Alanine

Alanine is synthesized by alanine aminotransferases using carbon from pyruvate and nitrogen derived from glutamate. Although alanine contributes to major cancer growth pathways, its role in cancer is still up for debate. Thus far, the alanine biosynthetic pathway has been connected to cancer proliferation and the secretion of alanine by pancreatic stromal cells is used in the TCA cycle [133].

### 9.2. Aspartate

Aspartate is linked to cancer growth and proliferation in a number of ways. Produced from OAA and glutamate-derived nitrogen, aspartate is crucial for the transfer of electrons from the cytosol to the mitochondria via the malate-aspartate shuttle (MAS). In the only irreversible step of the MAS, aspartate is exchanged for cytosolic glutamate and a proton by the aspartate-glutamate carrier (AGC) to provide electrons for OXPHOS. Since the concentration of aspartate in the plasma is low, cancer cells rely on the biosynthesis of aspartate by aspartate aminotransferase in the mitochondria [133,134,135]. Thus, aspartate is a limiting metabolite for many tumors in hypoxic conditions and cancer cells may display differential expression of AGC and aspartate aminotransferase to overcome this limitation, although more research is needed. Aspartate is also necessary for the synthesis of nucleotides and is a source of NADPH, connecting it in multiple ways to both cancer cell growth and survival [133].

### 9.3. Asparagine

Asparagine is another NEAA necessary for cancer cell growth under certain conditions. Specifically, when glutamine is depleted, asparagine is necessary for protein synthesis through its restoration of glutamine production [133,136,137]. Asparagine stabilizes glutamine synthetase (GLUL), which is the rate limiting enzyme in the conversion of glutamate to glutamine. Additionally, asparagine functions as an exchange factor that is needed for the uptake of amino acids required for the activation of mTOR signaling [133]. 

### 9.4. Cysteine

Cysteine is used by cancer cells as a carbon source, with increased cysteine bioavailability acting as a stimulus for metabolic reprogramming. When there is no limit on cysteine uptake, its contribution to cell growth and proliferation mainly occurs through cysteine catabolism [138]. Cysteine catabolism results in the production of organic compounds, such as pyruvate, α-glutarate, α-ketobutyrate, serine, propionyl-CoA, succinate, and acetyl-CoA to supply the TCA cycle, intermediates for fatty acid and protein synthesis, and hydrogen sulfide. Hydrogen sulfide can be used to donate electrons to the electron transport chain, and thus, is connected to ATP production. Hydrogen sulfide also acts as a signaling molecule in cancer that regulates cell proliferation [133]. 

Metabolic reliance on cysteine has been observed in several cancer types and concurrently involves the upregulation of cysteine catabolism and cysteine synthesis, as well as upregulation of the expression of cysteine transporters. Specifically, two enzymes involved in cysteine catabolism, cystathionine β-synthase (CBS) and cystathionine γ-lyase (CSE), are often upregulated. Increased expression of CBS and CSE is linked to enhanced rates of proliferation in cancer cells and is controlled by PI3K/Akt and Wnt pathways, respectively. CBS and CSE also play a role in cysteine synthesis, which occurs through the transsulfuration pathway (TSP) from methionine and serine [138]. Therefore, cysteine bioavailability is regulated by 1C metabolism. With high concentrations of cysteine, cancer cells diminish the need for cysteine biosynthesis through 1C metabolism and can instead utilize serine and methionine in the generation of 1C metabolites important for cell proliferation.

### 9.5. Proline

Proline is an important component of proteins, especially collagen, as its cyclic shape allows for a variety of protein structures. It is synthesized from glutamate and degraded by proline dehydrogenase. Both the biosynthetic and degradation pathways are regulated by Myc, giving proline context in the field of oncogenic signaling. Catabolism via proline dehydrogenase can promote cancer cell survival and have a tumor-suppressive function, depending on the type of tumor and the conditions of the tumor microenvironment. Proline is also a limiting factor for protein synthesis in some types of tumors [133]. Additional research is necessary to fully determine the role proline plays in cancer progression.

## 10. Branched Chain Amino Acid (BCAA) Metabolism

Branched chain amino acids (BCAAs) include the essential amino acids valine, leucine, and isoleucine and they play an important role in tumor cell growth and proliferation [139]. BCAAs function as nitrogen donors to produce nucleotides, can be incorporated into proteins, and can be broken down to produce important cell metabolites, such as glutamate, associating them with metabolic pathways critical for cancer progression [140]. Reprogrammed BCAA metabolism is involved in several types of cancer, including PDAC [141], glioblastoma [142], chronic myeloid leukemia (CML) [143], and endometrial cancer [144]. Since BCAAs are essential amino acids, they must be obtained from the diet before they can be utilized by tumors directly or degraded to other important metabolites. Though BCAA metabolic reprogramming in cancer is universal, the exact mechanisms are context and tumor dependent, with some cancers favoring direct BCAA usage and others favoring BCAA degradation.

**Figure 11:**
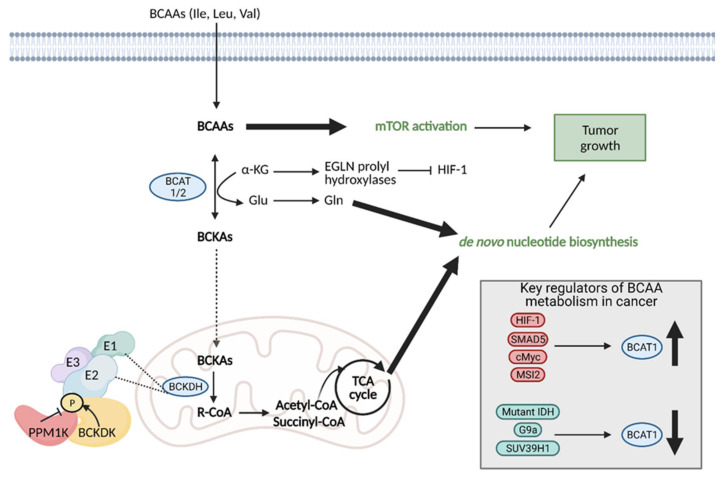
Reprogramming of branched chain amino acid (BCAA) metabolism in cancer. Branched chain amino acids (BCAAs), including isoleucine (Ile), leucine (Leu), and valine (Val), can be transported into the cell where they can directly activate mTOR signaling for tumor growth. They can also be converted to branched chain α-keto acids (BCKAs) via cytosolic branched chain amino acid transaminase 1 (BCAT1) or mitochondrial BCAT2 in a reversible reaction. BCAT1 overexpression results in increased BCAA catabolism, which is typical in cancer and is upregulated by several molecules (HIF-1, SMAD5, cMyc, MSI2), although some cancers favor the reverse reaction. The conversion of BCAAs to BCKAs generates glutamate, which can be used for de novo nucleotide biosynthesis. BCKAs can be further degraded in the mitochondria to acetyl CoA and succinyl CoA to power the TCA cycle and de novo nucleotide biosynthesis to support cancer proliferation. α-KG; alpha-ketoglutarate, BCKDH; branched-chain alpha-keto acid dehydrogenase complex, Glu; glutamate, Gln; glutamine, HIF-1; hypoxia-inducible factor 1, IDH; isocitrate dehydrogenase, MSI2; Musashi2, mTOR; mammalian target of rapamycin; PPM1K; Mg2+/Mn2+- dependent 1 K protein phosphatase, R-CoA; R-coenzyme A. Figure created with BioRender.com (accessed on 26 March 2021).

Regardless of the mechanism preferred, the enzymes involved in the first step of BCAA degradation are upregulated in cancer and they are reversible. This includes cytosolic branched-chain aminotransferase (BCAT1) and mitochondrial branched-chain aminotransferase (BCAT2), which convert BCAAS into branched-chain α-keto acids (BCKAs) by transferring the amino group to α-KG to generate glutamate or the reverse reaction [140]. Specifically, BCAT1 expression has been implicated in cancer growth, with suppression of BCAT1 limiting proliferation. BCAT1 expression is regulated by a number of molecules, including upregulation by HIF-1, SMAD5, c-Myc, and Musashi2 (MSI2), and downregulation by mutant IDH, and histone modifiers, G9a, and SUV39H1 (Figure 11) [139]. BCAT expression is particularly important because it controls the balance between BCAAs and BCKAs, and the balance between α-KG and glutamate in the cell. 

In tumors that favor direct BCAA usage and low BCAA catabolism, such as breast cancer and leukemia, BCAT1 catalyzes the reamination of BCKAs to BCAAs, resulting in the accumulation of BCAAs. High levels of BCCAs then promote tumor growth by activating mTORC1 and the mTOR downstream signaling pathway [139,140].

**Figure 12:**
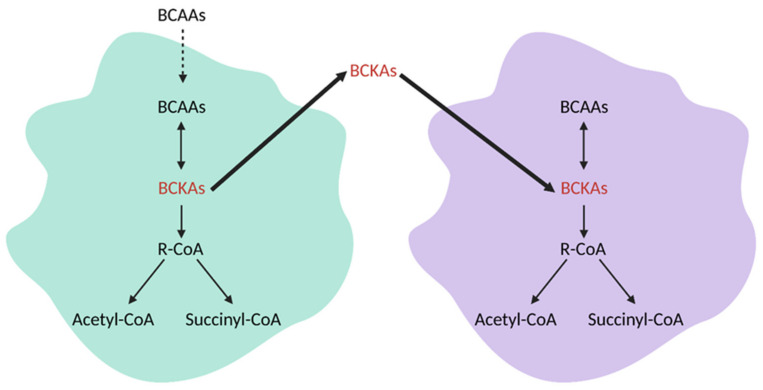
Branched chain amino acid (BCAA) metabolism intercommunication in the tumor microenvironment. Branched chain α-keto acids (BCKAs) produced by one cell in the tumor microenvironment can be taken up and utilized by a neighboring cell. The neighboring cell can then degrade the BCKAs to acetyl-CoA or succinyl CoA or convert them back to BCAAs depending on metabolic needs. R-CoA; R-coenzyme A. Figure created with BioRender.com (accessed on 26 March 2021).

Other tumors, such as gliomas, rely on increased BCAA catabolism via BCAT1 for growth. BCAA degradation restricts α-KG, which diminishes the activity of α-KG-dependent dioxygenases. Low α-KG levels attenuate the activity of a specific group of α-KG-dependent dioxygenases, termed EGLN prolyl hydroxylases. EGLN prolyl hydroxylases block HIF-1 activation by tagging it for proteasomal degradation, and thus, decreased α-KG levels impair this group of enzymes and cause HIF-1 activation. HIF-1 activation allows cancer cells to survive in hypoxic conditions through the activation of target genes. Additionally, increased BCAA catabolism results higher production of glutamate, which can be used for DNA and protein synthesis to facilitate cancer cell proliferation [139]. Tumors can also have a mix of cells, with some populations performing little BCAA catabolism and others displaying increased amounts of BCAA catabolism. This is accomplished in a manner similar to that of the reverse Warburg Effect, with BCKAs produced by one cell able to be utilized by a neighboring cell for BCAA reamination (Figure 12) [139]. 

After degradation, BCKAs then undergo decarboxylation via an irreversible reaction by the branched-chain α-keto acid dehydrogenase (BCKDH) complex, located in the mitochondria. The activity of the BCKDH complex is regulated by pair of enzymes, branched chain keto acid dehydrogenase kinase (BCKDK) and Mg2+ / Mn2+- dependent 1 K protein phosphatase (PPM1K), which are upregulated in many tumors. BCKDK overexpression suppresses BCKA decarboxylation but enhances tumor growth by increasing BCAA levels and activating the MAPK pathway [145,146]. When BCKDK activity is low, the BCKDH complex proceeds and BCKAs are ultimately metabolized to acetyl CoA and succinyl-CoA, which can be fed into the TCA cycle, making it an energy source for tumors. This pathway is especially important for growth in PDAC tumors that exhibit an increased reliance of BCKAs in times of BCAA deprivation [139].

## 11. Regulation of Apoptosis by Metabolism

While healthy cells typically undergo programmed cell death or apoptosis during regular progression of the cell cycle, cancer cells evade apoptosis to enhance growth, proliferation, and survival under hypoxic conditions. Many of their apoptosis evasion mechanisms are linked to metabolic reprogramming [147].

**Figure 13:**
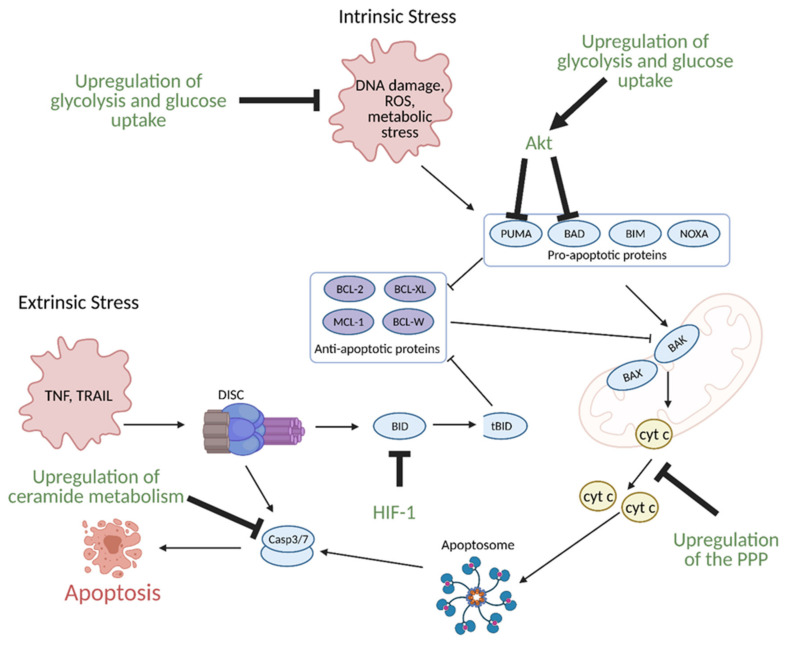
Regulation of apoptosis through metabolism in cancer. Apoptosis can be induced via intrinsic or extrinsic stress. Intrinsic stress results in the activation of pro-apoptotic proteins that then activate B-cell lymphoma 2 (Bcl-2)-associated X protein (BAX) and Bcl-2-homologous antagonist killer (BAK) in the mitochondria to facilitate cytochrome c (cyt c) release via mitochondrial outer membrane polarization. Cytochrome c release results in the formation of the apoptosome, which leads to the activation of caspase 3 (Casp3) and caspase 7 (Casp7). Casp3 and Casp7 then induce apoptosis. Extrinsic stress results in the formation of the Death Inducing Signaling Complex (DISC), which can directly activate apoptosis or trigger intrinsic apoptosis via BH3 interacting-domain death agonist (BID) cleavage to truncated BID (tBID). Cancer avoids apoptosis through multiple metabolism-mediated mechanisms, including reduction of reactive oxygen species (ROS) via the upregulation of glycolysis and glucose uptake, inhibition of pro-apoptotic proteins via growth factor signaling, inhibition of cyt c release via upregulation of the pentose phosphate pathway (PPP), inhibition of BID cleavage via hypoxia-inducible factor 1 (HIF-1), and inhibition of effector caspases via the upregulation of ceramide metabolism. Akt; protein kinase B, BAD; Bcl-2 associated death promoter, BIM; Bcl-2-like protein 11, BCL-W; Bcl-like protein 2, BCL-XL; Bcl-extra-large, MCL-1; myeloid-cell leukemia 1, NOXA; phorbol-12-myristate-13-acetate-induced protein 1, PUMA; p53 upregulated modulator of apoptosis. Figure created with BioRender.com (accessed on 26 March 2021).

Glucose metabolism, specifically enhanced glycolysis, is one of the main hallmarks of metabolic reprogramming in cancer. Glucose metabolism is linked to the evasion of apoptosis in several ways. Many of the same signaling molecules involved in the upregulation of glycolysis are also involved in the suppression of apoptosis. For example, the hypoxic tumor microenvironment induces expression of aforementioned HIF-1, which in turn, leads to overexpression of glycolytic enzymes. Upregulation of glycolysis and glucose uptake are linked to resistance to apoptosis as increased glycolysis and glucose uptake prevents oxygen related damage to the cell, and thus, results in decreased apoptosis. HIF-1 also directly induces resistance to apoptosis via suppression of pro-apoptotic B cell lymphoma 2 (BCL-2) family protein BH3 interacting-domain death agonist (BID) (Figure 13) [148,149].

Akt, another regulator of glycolysis in cancer cells, blocks apoptosis via suppression of two pro-apoptotic BCL-2 family proteins, p53 upregulated modulator of apoptosis (PUMA) (Figure 13) and glycogen synthase kinase 3 (GSK-3). Additional molecules involved in glucose metabolism that are involved in the suppression of apoptosis include BCL2 associated agonist of cell death (BAD), TP53-Induced Glycolysis and Apoptosis Regulator (TIGAR), and cytochrome c. BAD is phosphorylated by Akt, protein kinase A (PKA), and c-Jun N-terminal protein kinase (JNK), which are regulated by glycolysis and cell growth signaling [147,150]. The phosphorylation of BAD is a modification that makes it unable to promote apoptosis and it instead contributes to upregulation of glycolysis by activating glucokinase and PFK1 [147]. Cytochrome c activates the intrinsic apoptotic pathway but undergoes modification by glucose metabolism. Enhanced pentose phosphate pathway flux in cancer cells results in increased production of NADPH, which inhibits cytochrome c activity by keeping it in its inactive reduced form [151]. TIGAR is normally activated by p53 in response to DNA damage and stress and suppresses glycolysis in favor of the pentose phosphate pathway. This leads to decreased intracellular ROS levels via increased production of antioxidant NADPH, therefore leading to the suppression of ROS induced apoptosis [147]. As a suppressor of glycolysis, however, the exact role of TIGAR as a tumor enhancer or suppressor is controversial. As a p53 inducible gene, it is most likely a tumor suppressor. Since p53 is often mutated or inactive in cancer, it is suggested that TIGAR may be induced by a different set of genes independent of p53 in these types of cancer to function as a tumor enhancer [147]. Thus, the reprogramming of glucose metabolism in cancer enhances cancer progression not only via pathways promoting growth, but also via mechanisms that avoid cell death and enhance survival. 

Lipid metabolic reprogramming in cancer is also linked to the regulation of apoptosis, specifically via pathways involving sphingolipid production [147]. Ceramide, produced from serine and palmitoyl CoA, is considered the central metabolite of sphingolipid metabolism and it can be synthesized via three different pathways, including the de novo pathway, the sphingomyelinase (SMase) pathway, and the salvage pathway [152]. Ceramide can be metabolized to a variety of sphingolipids, including sphingomyelins, glycosphingolipids, and gangliosides. Each type of sphingolipid plays a specific role in the context of cancer cell survival, with some promoting apoptosis and others promoting survival. Cancer cells typically demonstrate an upregulation in the production of sphingolipids that support the evasion of apoptosis and promote cell survival [153]. This includes upregulation of ceramide metabolism via increased activities of glucosylceramide synthase (GCS), sphingomyelin synthase (SMS), ceramide kinase (CERK), acid ceramidase (AC) and/or sphingosine kinase (SPHK), all of which catalyze the production of sphingolipids with pro-survival functions [154]. For example, the hydrolysis of ceramide to sphingosine by AC, and subsequent phosphorylation by SPHK to produce sphingosine-1-phosphate (S1P) promotes cell survival as S1P interacts with its receptor S1PR to activate oncogenic signaling. This includes apoptosis suppression via inhibition of caspase 3 and induction of cell proliferation via activation of peroxisome proliferator-activated receptors (PPARs) and target genes. Thus, cancer cells exhibit overexpression of AC and SPHK to promote S1P production, apoptosis evasion, and cell growth [152,154].

## 12. Role of Non-Coding RNAs in Cancer Cell Metabolism

Although the role of oncogenes, transcription factors, and other downstream signaling molecules has been widely established in mechanisms of cancer metabolic reprogramming, non-coding (nc) RNAs are emerging as important players. As ncRNAs were initially considered to lack biological function because they do not encode proteins, they play a role in cancer progression by regulating enzymes and pathways involved in the metabolic reprogramming of cancer cells. This regulation primarily occurs through glucose, glutamine, and lipid metabolism and involves two types of ncRNA, long-chain non-coding RNA (lncRNA) and microRNA (miRNA) [155]. 

Our recent understanding that ncRNAs can affect a cell has also implicated deregulated ncRNA expression in cancer development and progression. Amplification of chromosomal regions that encode for oncogenic ncRNAs are found in cancer [156]. Some of these ncRNAs directly target metabolic processes. There are several enzymes related to glucose metabolism that are regulated by ncRNAs in cancer. HK2 expression, for example, is downregulated by miR-199a-5p and miR-125b, with lower expression of these ncRNAs corresponding with enhanced growth [155]. In breast cancer, GLUT1 and PK expression are downregulated by the secretion of vesicles containing miR-122, which decreases glucose uptake in non-tumor cells to increase nutrient availability in pre-metastatic cells and promote metastasis [157]. LDHA activity is enhanced by Lnc-IGFBP4-1 to promote metastasis and ATP production [158]. Lipid and glutamine metabolic reprogramming are also influenced by ncRNAs, though most of the ncRNAs involved in these pathways thus far suppress cancer metastasis. Importantly, the PI3K/Akt/mTOR pathway is also regulated by ncRNAs. For example, miR-149-5p, activated by circulating endogenous RNA circNRIP1, stimulates the PI3K/Akt/mTOR pathway to promote cancer cell growth. The PI3K/Akt/mTOR pathway can also be activated by LINC00963 via PGK1 ubiquitination blockage and by miR-384 via upregulation of pleiotrophin and lipogenic genes [155]. The variety of ncRNAs that affect metabolic processes and upstream oncogenic signaling pathways demonstrates the growing importance of ncRNAs in the field.

## 13. Regulation of Cancer Growth via Tumor-Host Cell Metabolic Interactions

The reprogramming of metabolic pathways in cancer involves not only tumor cells themselves but also interactions between tumor cells and host cell populations [159]. The tumor microenvironment is dense and includes fibroblasts, macrophages, mesenchymal stem cells, endothelial cells, and immune cells in addition to cancer cells [160]. Heterocellular metabolic interactions between these populations in the tumor microenvironment work cohesively to support tumor growth and proliferation. With several different cell populations taking up residence, the tumor microenvironment faces many challenges and limiting factors for survival, including physical pressure, oxidative stress, nutrient deprivation, competition, and immune surveillance. To surmount these challenges and achieve tumor progression, tumor cells take advantage of the diverse microenvironment and engage in complex crosstalk with surrounding cells via nutrient sharing and metabolic symbiosis, competition, and the use of metabolites as signaling molecules [159]. 

Nutrient sharing and metabolic symbiosis are common in multiple types of tumors and most significantly involve lactate. Glucose-derived lactate plays a multifaceted role in that it can originate in hypoxic cancer cells and feed nearby tumor cells, originate in fibroblasts and feed tumor cells, or originate in tumor cells and feed mesenchymal stem cells and fibroblasts. It can also act as a signaling molecule and affect immune cell populations, either by polarizing macrophages toward a tumor-associated macrophage fate or inhibiting antitumor T cells [161]. This mode of symbiotic metabolism is observed in multiple types of cancer, including lung cancer [162], breast cancer [163], PDAC [164], and colon cancer [165]. Nutrient sharing of amino acids and other metabolites between fibroblasts and cancer cells also occurs. One example is the secretion of alanine by pancreatic cancer-associated fibroblasts in response to interaction with pancreatic cancer cells [166]. Alanine is taken up by the pancreatic cancer cells and used for macromolecule biosynthesis. The sharing of molecules between different populations of cells in the tumor microenvironment allows for cancer cell growth by maximizing use of available nutrients for energy and macromolecule biosynthesis, providing alternative energy sources, and influencing immune cell populations.

Nutrient recycling between tumor cells and fibroblasts is also important for tumor growth. The main function of fibroblasts is to produce and secrete extracellular matrix, which increases the diversity of macromolecules surrounding the tumor cells [159]. The macromolecules produced by fibroblasts, such as collagen, can be taken up by cancer cells via macropinocytosis and released into the cytosol for use in metabolic processes. Upregulation of micropinocytosis is often observed in PDAC tumors to compensate for their lack of amino acids [161].

While many symbiotic tumor-host cell metabolic interactions contribute to tumor growth, competition between tumor and host cells for nutrients also significantly influences cancer progression [167]. In order to carry out biosynthetic and bioenergetic activities, immune, tumor and stromal cells must all compete for nutrients. Immune cells are not adapted for competition, which leads to tumor growth via a loss of anti-tumor immune surveillance [159]. Glucose availability is a crucial factor involved as cancer cells, dendritic cells, macrophages, T cells, and B cells are highly glycolytic. Glucose depletion in the tumor microenvironment observed in many cancer types can, therefore, diminish the anti-tumor activity of immune cell populations and lead to cancer progression [160]. The metabolism of amino acids, lipids, and one-carbon units by cancer cells can also influence immune cell metabolism. For example, serine and arginine are important for T cell expansion, survival, and antitumor activity [160]. In this respect, the metabolic needs of T cells are similar to cancer cells, and depletion of amino acids from T cells via competition with cancer cells may contribute to suppression of an anti-cancer immune response. 

It has been suggested that treatment with a ketogenic diet impairs the metabolism of cancer cells and restores immune cell function. High in fat and protein and low in carbohydrates, a ketogenic diet results in less carbohydrate uptake, and consequently, leads to cancer cell starvation and cell death [168]. A ketogenic diet also results in the production of ketone bodies, which are unable to be metabolized in many types of cancers [169]. This creates an unfavorable metabolic environment for cancer cell proliferation, and instead, allows host cell populations to flourish [168]. Ketone bodies can be anti-inflammatory, affect glucose metabolism, mitochondrial metabolism, and amino acid metabolism of cancer cells, act as signaling molecules that increase the expression of tumor suppressive molecules, and disrupt tumor angiogenesis [168,170], making a ketogenic diet an important potential anti-cancer therapy. However, the efficacy of ketogenic diet in cancer treatment is dependent on tumor type, as different tumor types exhibit distinct metabolic reprogramming [168].

## 14. Anti-Cancer Drugs That Target Metabolism

Increased knowledge and understanding of metabolic reprogramming have led to the development of cancer therapies that target various aspects of metabolism. Pharmacological targeting of these pathways has the potential to significantly reduce cancer growth and proliferation. This is evident by the large number of drugs in development that target glucose metabolism, lipid metabolism, one-carbon metabolism, and various growth pathways (Table 1). With further elucidation of the mechanisms of metabolic reprogramming in cancer cells, there is the potential for improvement of current therapies and the discovery of new and enhanced metabolism-targeting therapies.

## 15. Conclusions

Much progress has been made in the field of cancer metabolism since Warburg’s initial observations about 100 years ago. While cancer cells were previously thought to perform only aerobic glycolysis, it is now evident their growth and proliferation is dependent on the reprogramming of a large number of pathways, such as the TCA cycle, the PPP, lipogenesis, 1C metabolism, and BCAA metabolism, as well as the utilization of alternate substrates, conferring them with metabolic plasticity. Altered flux through metabolic pathways is coordinated by many different genes and regulatory molecules that work together to support enhance growth and proliferation. Crosstalk between many of these pathways is evident, including that between glycolysis and the PPP and the regulation of apoptosis through metabolism. Understanding the complex nature of altered metabolic flux in cancer cells and its context dependent nature is crucial to the development and application of new therapies. 

## Figures and Tables

**Table 1:** Anti-cancer drugs that target metabolism.

Drug	Target	State of Development	References
2-deoxyglucose (2-DG)	Hexokinase 2 (HK2)	Phase II	[171,172]
3-bromopyruvate (3BP)		Phase I	[173,174,175]
Lonidamine		Phase II	[176,177]
Genistein-27		Preclinical	[178]
Benserazide		Preclinical	[179]
Resveratrol		Phase I	[180,181]
Astragalin		Preclinical	[182]
Chrysin		Preclinical	[183]
Silybin	Glucose transporters (GLUTs)	Phase I	[184]
Cytochalasin B		Preclinical	[185]
Phloretin		Preclinical	[186,187]
Fasentin		Preclinical	[188]
STF-31		Preclinical	[189]
WZB117		Preclinical	[190,191]
Ritonavir		Preclinical	[192]
Koningic acid	Glyceraldehyde-3-phosphate dehydrogenase (GAPDH)	Preclinical	[193,194]
Iodoacetate		Preclinical	[195]
CPI-613	Pyruvate dehydrogenase (PDH)/α-ketoglutarate dehydrogenase	Phase III	[196,197,198]
Dichloroacetate (DCA)	Pyruvate dehydrogenase kinase (PDK)	Phase I	[199,200,201,202]
Mitaplatin (cisplatin and DCA fusion)		Phase I	[203]
Oxamate	Lactate dehydrogenase (LDHA)	Preclinical	[204]
FX11		Preclinical	[205]
α-Cyano-4-hydroxycinnamic acid		Preclinical	[206]
Cinnamate	Monocarboxylate transporters (MCTs)	Preclinical	[207]
AZD3965		Phase I	[208,209]
Afuresertib	PI3K/Akt	Phase I	[210,211,212]
Uprosertib		Phase I	
Ipatasertib		Phase I	
Sorafenib		Phase II	[213]
Metformin	Complex I, oxidative phosphorylation (OXPHOS)	Preclinical and clinical studies	[214]
Phenformin		Preclinical and clinical studies	[215]
Lonidamine	Complex II (OXPHOS)	Phase II	[216,217,218,219]
Atovaquone	Complex III (OXPHOS)	Early Phase I	[220]
Arsenic trioxide	Complex IV (OXPHOS)	Phase III	[22,221]
Nitric oxide		Preclinical and clinical studies	[222]
BPTES	Glutaminase (GLS1)	Preclinical	[223,224]
CB-839		Phase II	[225,226,227]
JHU-083		Preclinical	[228]
Dehydroepiandrosterone (DHEA)	Glucose-6-phosphate dehydrogenase (G6PD)	Phase I	[229,230]
Polydatin		Preclinical	[231]
6-aminonicotinamide (6-AN)	6-phosphogluconate dehydrogenase (6GPD)	Preclinical	[232]
Etomoxir	Carnitine palmitoyl transferase 1 (CPT1)	Retired from phase II clinical trials for diabetes and heart failures	[233,234]
TVB-2640	Fatty acid synthase (FASN)	Phase II	[235,236]
Cerulenin		Preclinical	[237]
Orlistat		Preclinical	[238]
GSK2194069		Preclinical	[236]
Triclosan		Discontinued for safety issues	[239,240]
Fasnall		Preclinical	[241]
SB-204990	ATP-citrate lyase (ACLY)	Preclinical	[242]
Soraphen A	Acetyl-CoA carboxylase (ACC)	Preclinical	[243,244]
BZ36	Stearoyl-CoA desaturase (SCD)	Preclinical	[245]
A939572		Preclinical	[246]
Fatostatin	Sterol regulatory element-binding protein (SREBP)	Preclinical	[247]
Betulin		Preclinical	[248]
Triacscin C	Acetyl-CoA synthase (ACS)	Preclinical	[249]
Statins	3-hydroxy-methylglutaryl-CoA reductase (HMGCR)	Preclinical and clinical studies	[250,251]
AG-120 (ivosidenib)	Mutant isocitrate dehydrogenase 1 (IDH1)	Phase III	[252,253]
IDH305	Mutant IDH2	Phase II	[254,255,256]
BAY1436032	Mutant IDH1/2	Phase I	[257,258,259]
FT-2102		Phase II	[260]
AG-221 (enasidenib)		Phase III	[261,262,263]
AG-881		Phase III	[264,265]
AGF347	Serine hydroxymethyltransferase 1/2 (SHMT1/2)	Preclinical	[266]
LY345899	Methylene tetrahydrofolate dehydrogenase 2 (MTHFD2)	Preclinical	[267]
Carolacton	MTHFD1/2	Preclinical	[268]
LY231514/MTA/pemetrexed	Dihydrofolate reductase (DHFR), thymidylate synthase (TS), glycinamide ribonucleotide formyltransferase (GARFT)	Phase IV	[269,270,271]
Amethopterin/MTX/methotrexate	TS, DHFR	Phase IV	[272,273]
Capecitabine	TS	Phase IV	[274,275,276]
5-Fluorouracil		Phase III	[277]
6-Mercaptopurine	Phosphoribosyl pyrophosphate amidotransferase (PPAT)	Phase III	[278,279]
6-Thioguanine		Phase III	[280,281]

## Data Availability

No new data were created or analyzed in this study.
