# Correction: Schiliro, C.; Firestein, B.L. Mechanisms of Metabolic Reprogramming in Cancer Cells Supporting Enhanced Growth and Proliferation. *Cells* 2021, *10*, 1056

**DOI:** 10.3390/cells11223593

**Published:** 2022-11-14

**Authors:** Chelsea Schiliro, Bonnie L. Firestein

**Affiliations:** 1 Cell and Developmental Biology Graduate Program and Department of Cell Biology and Neuroscience, Rutgers, The State University of New Jersey, 604 Allison Road, Piscataway, NJ 08854, USA; 2 Department of Cell Biology and Neuroscience, Rutgers, The State University of New Jersey, 604 Allison Road, Piscataway, NJ 08854, USA

The authors wish to make the following changes to their paper [1]. In the original publication, there was a mistake in the legend for Figure 6. We inadvertently forgot to cite the paper from which the figure was modified. The correct legend appears below.

**Figure 6**. Lipid metabolic reprogramming in Cancer. An overview of lipid metabolic pathways and how they are modified in cancer. (**a**). Tumor cells take up fatty acids (FAs) using multiple trans-porters, including CD36, FA binding proteins 1-6 (FABP1-6), and a low-density lipoprotein receptor (LDLR) for low-density lipoproteins (LDL). These free FAs then become a part of the cellular FA pool where they can enter the citric acid (TCA) cycle and contribute to lipid formation. The upregulation of FA uptake in cancer occurs through hypoxia-inducible factor (HIF-1)-induced FABP1-6 over-expression. (**b**). The upregulation of lipogenesis and cholesterol biosynthesis is achieved through sterol regulatory element binding protein (SREBP) activation. SREBP1 activation induces the ex-pression of lipogenesis genes, while SREBP2 activation induces the expression of cholesterol bio-synthesis genes. (**c**). Fatty acid oxidation (FAO) can be upregulated by cMyc, depending on the cancer type as a means to counteract oxidative stress. ACC1/2: acetyl-CoA carboxylase 1/2, ACLY: ATP citrate lyase, ACS: acyl-CoA synthetase, α-KG: alpha-ketoglutarate, CoA: coenzyme A, CPT1: carnitine palmitoyltransferase 1, FADS: FA desaturases, FASN: fatty acid synthase, FPP: farne-syl-pyrophosphate, GLUT1: glucose transporter 1, HMG-CoA: hydroxy-methylglutaryl-CoA, HMGCS: hydroxy-methylglutaryl-CoA synthase, HMGCR: hydroxy-methylglutaryl-CoA reduc-tase, LD: lipid droplets, MUFA: monounsaturated fatty acids, PUFA: polyunsaturated fatty acids, SCD1: stearoyl-CoA desaturase 1, SOAT: sterol O-acyltransferase. The figure is created with Bio-Render.com (accessed on 26 March 2021). This figure is modified from Figure 1 in [78].

New reference number 78 will be added to the reference list. All reference citations after reference 78 will be changed; numbers will increase by one, e.g., 78 will be 79, etc. The reference 78 in the reference list should be:

78. Bacci, M.; Lorito, N.; Smiriglia, A.; Morandi, A. Fat and Furious: Lipid Metabolism in Antitumoral Therapy Response and Resistence. *Trends Cancer* **2020**, *7*, 198–213.

The authors apologize for any inconvenience caused and state that the scientific conclusions are unaffected. This correction was approved by the Academic Editor. The original publication has also been updated.

## References

[1] Schiliro C., Firestein B.L. (2021). Mechanisms of Metabolic Reprogramming in Cancer Cells Supporting Enhanced Growth and Proliferation. Cells.

